# A Novel Role for *Dbx1*-Derived Cajal-Retzius Cells in Early Regionalization of the Cerebral Cortical Neuroepithelium

**DOI:** 10.1371/journal.pbio.1000440

**Published:** 2010-07-27

**Authors:** Amélie Griveau, Ugo Borello, Frédéric Causeret, Fadel Tissir, Nicole Boggetto, Sonia Karaz, Alessandra Pierani

**Affiliations:** 1 CNRS-UMR 7592, Program of Development and Neurobiology, Institut Jacques Monod, Université Paris Diderot, Paris, France; 2 Developmental Neurobiology, Institute of Neuroscience, Université catholique de Louvain, Brussels, Belgium; University of North Carolina, United States of America

## Abstract

Patterning of the cortical neuroepithelium occurs at early stages of embryonic development in response to secreted molecules from signaling centers. These signals have been shown to establish the graded expression of transcription factors in progenitors within the ventricular zone and to control the size and positioning of cortical areas. Cajal-Retzius (CR) cells are among the earliest generated cortical neurons and migrate from the borders of the developing pallium to cover the cortical primordium by E11.5. We show that molecularly distinct CR subtypes distribute in specific combinations in pallial territories at the time of cortical regionalization. By means of genetic ablation experiments in mice, we report that loss of septum *Dbx1*-derived CR cells in the rostromedial pallium between E10.5 and E11.5 results in the redistribution of CR subtypes. This leads to changes in the expression of transcription factors within the neuroepithelium and in the proliferation properties of medial and dorsal cortical progenitors. Early regionalization defects correlate with shifts in the positioning of cortical areas at postnatal stages in the absence of alterations of gene expression at signaling centers. We show that septum-derived CR neurons express a highly specific repertoire of signaling factors. Our results strongly suggest that these cells, migrating over long distances and positioned in the postmitotic compartment, signal to ventricular zone progenitors and, thus, function as modulators of early cortical patterning.

## Abstract 2

Patterning of the cerebral cortex during embryogenesis depends not only on passive diffusion of morphogens but also on signal delivery by Cajal-Retzius neurons that migrate over long distances.

## Author Summary

Patterning of the cerebral cortex occurs early during embryonic development in response to secreted molecules or morphogens produced at signaling centers. These morphogens establish the graded expression of transcription factors (TFs) in progenitor cells and control the size and positioning of cortical areas in the postnatal animal. CR cells are among the earliest born cortical neurons and play a crucial role in cortical lamination. They are generated at signaling centers and migrate over long distances to cover its entire surface. We show that three different CR subtypes distribute in specific proportions in cortical territories. Genetic ablation of one subpopulation leads to a highly dynamic redistribution of the two others. This results in defects in expression of transcription factors and in progenitor cell proliferation, which correlate with the resulting changes in the size and positioning of cortical areas. Given our additional evidence that CR subtypes express specific repertoires of signaling factors, the ablation phenotypes point to a novel early role for CR cells as mediators of cortical patterning and suggest that CR cells are able to signal to progenitor cells. Our data thus add to the conventional model that morphogens act by passive diffusion and point to a strategy of morphogen delivery over long distance by migrating cells.

## Introduction

Patterning is defined as the process by which an equipotent field of cells proliferates and organizes into a complex spatial arrangement of distinct cell types in response to positional information [1]. The coordinated growth and patterning of both adjacent and distant developing tissues along the anteroposterior and dorsoventral axes is crucial to ensuring the harmonious construction of a functional body. Patterning mechanisms in early embryos have been studied for many years. These studies have led to the identification of signaling centers and their secreted molecules, some of which mediate their function in a dose-dependent manner, such as Fgfs, Bmps, Shh, RA, or Wnts, as well as their antagonists. Morphogens appear to affect patterning (growth and cell fate) over long distances. In the developing cerebral cortex, they have been shown to control the graded expression of transcription factors (TFs) in progenitors along the rostrocaudal (RC) and mediolateral axes of the neuroepithelium [2]–[5]. Although a major effort has been directed towards understanding how signaling information travels from the source through the surrounding tissues in both vertebrates and *Drosophila* [6],[7], how this leads to graded expression of transcription factors and coordinates growth and cell fate over long distance is still an unresolved issue.

The cerebral cortex has a laminar organization in which earlier and later born neurons accumulate according to an inside-out sequence. It is divided into areas which serve distinct functions ranging from motor and sensory to cognitive processing. These territories have a specific size and are positioned at precise spatial coordinates relative to each other. The achievement of such a highly complex architecture requires the exquisite orchestration of the proliferation of progenitors, the spatio-temporal generation of distinct cell types, and the regulation of their migration and final positioning. Commitment to a cortical regional phenotype occurs during early stages of development, between E10.5 and E12.5 [8]–[10]. The Pax6, Sp8, CoupTF1, and Emx2 transcription factors have been shown to regulate the early regionalization of the cortical neuroepithelium and to play a crucial role in controlling the size and position of cortical areas in the postnatal cortex [2]–[5].

Cajal-Retzius (CR) cells are among the first neurons to be generated in the embryonic telencephalon. They start invading the preplate at E10.0–E10.5 in mice [11]–[13] and are subsequently localized in the most superficial layer (marginal zone (MZ)/layer I) of the developing cortex until the first postnatal weeks. Their best documented function is to control the radial migration of neurons and the formation of cortical layers by secreting the extracellular glycoprotein Reelin (Reln) [14]–[16]. Although additional functions for CR cells have been proposed at late stages of development, such as the regulation of the radial glia phenotype [17],[18] and the development of hippocampal connections [19], their molecular properties and function during early corticogenesis remain elusive.

Genetic tracing experiments have disclosed at least three sites of origin of CR neurons at the borders of the developing pallium: the pallial-subpallial boundary (PSB or anti-hem) laterally, the pallial septum (also called retrobulbar area, commissural plate, medial-PSB) rostromedially, and the cortical hem caudomedially [20]–[22]. Hem-derived CR neurons have been shown to predominantly populate the caudomedial and dorsolateral pallium at E12.5 [22]. We have shown that cells expressing the homeodomain transcription factor Dbx1 at the septum and the PSB give rise to two molecularly distinct subtypes of CR cells that migrate over long distances from their origins to primarily populate the rostromedial and lateral developing pallium, respectively [20]. The choroid plexus and the thalamic eminence have also been suggested to generate CR neurons which invade caudoventral telencephalic regions [23]–[25].

In this report, we use genetic ablation to study the role of CR subpopulations in cortical development. We show that changing the dynamic distribution of molecularly distinct CR subtypes in pallial territories influences early cortical regionalization and postnatal arealization by controlling proliferation of progenitors within the ventricular zone (VZ). Our results show that septum-derived CR neurons express a highly specific repertoire of signaling factors and suggest that their secretion might be one of the mechanisms by which CR neurons from the postmitotic compartment contribute to cortical patterning.

## Results

### Molecularly Distinct CR Subtypes Are Distributed in Specific Combinations in Early Pallial Territories

Reln, p73 and Calretinin have been used alone or in combination as specific markers to define CR neurons [12],[13],[20],[26],[27]. Whereas Reln appears to be expressed by all CR cells, the expression of these three proteins do not overlap completely in the early preplate or during later stages of development within the MZ. We have previously shown that septum- and PSB-derived CR cells are molecularly distinct and that the former do not express one of the two Calretinin isoforms [20] (recognized by goat anti-Calretinin, Swant). At E11.5, p73 has been reported to be an early marker for CR neurons derived from the cortical hem, prior to their onset of Reln expression, whereas PSB-derived CR cells only express Reln at this stage [26],[27]. While additional genes have been shown to be expressed in CR neurons [25],[28],[29], these were also expressed by other preplate cell populations.

**Figure 1:**
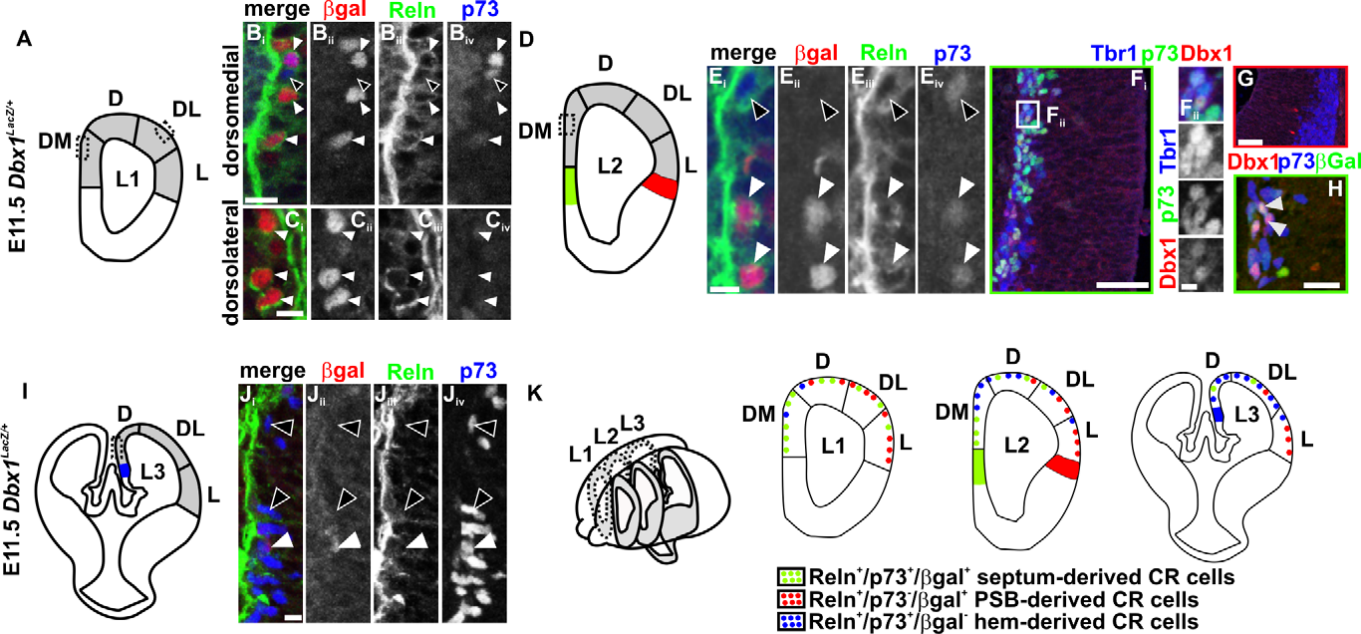
Molecularly distinct CR subtypes populate the rostral preplate. E11.5 coronal sections of *Dbx1^nlsLacZ/+^* telencephalons were immunostained for βgal, Reln and p73. At L1 levels (A–C), DM territories are mainly populated by septum-derived βgal^+^/Reln^+^/p73^+^ triple-labeled cells (white arrowheads, B_i_–B_iv_). Very few hem-derived βgal^−^/Reln^+^/p73^+^ cells can be detected (black arrowheads, B_i_–B_iv_). In the L and DL regions, βgal^+^ cells are only Reln^+^ (white arrowheads, C_i_–C_iv_). No Dbx1^+^ cells are detected at this level. At L2 levels (D–E), more βgal^−^/Reln^+^/p73^+^ cells are detected (black arrowheads, E_i_–E_iv_) compared to L1, positioned dorsally to βgal^+^/Reln^+^/p73^+^ cells (white arrowhead, E_i_–E_iv_) in DM regions. Dbx1^+^ cells were exclusively found at the septum (F_i_, F_ii_, and H) and at the PSB (G) (green and red domains, respectively, in D) by immunostaining. Dbx1 (red) is coexpressed with Tbr1 (blue) and p73 (green) (F_i_ and F_ii_) in postmitotic CR cells. Dbx1 at the PSB is expressed in progenitors (Tbr1^−^) (G). Co-labeling of Dbx1, p73 (blue), and βgal (green) at the septum in *Dbx1^nlsLacZ/+^* embryos (H). At caudal levels (L3, I–J), βgal^−^/p73^+^ cells (black arrowheads, J_i_–J_iv_) derived from the hem (blue domain) represent the majority of Reln^+^ cells in D regions. Scattered βgal^+^/Reln^+^/p73^+^ cells can be found in this domain (white arrowheads). (K) Schematic representation illustrating the distribution of CR cells subtypes in pallial territories at E11.5. In L1, septum-derived CR cells (Reln^+^/p73^+^/βgal^+^, green) are the main CR subtype in DM and D regions (and represent 95% to 60% of βgal^+^/Reln^+^ progressing from DM to D), whereas PSB-derived CR cells (Reln^+^/p73^−^/βgal^+^, red) invade the DL and L territories. Less than 10% of hem-derived CR cells (Reln^+^/p73^+^/βgal^−^, blue) were found on such rostral sections. In L2, septum and hem-derived CR cells are mixed in DM and D regions and represent 60–70% and 20–30% of CR cells, respectively, in DM regions. In L3, hem-derived CR cells are the main population in D (85–95% of CR cells) and DL territories, whereas PSB-derived CR cells are in the L regions all along the RC axis and represent 50–60% of lateral cells. Scale bars: 50 µm (F_i_ and G), 20 µm (H), 10 µm (B_i_–C_iv_, E_i_–E_iv_, F_ii_, J_i_–J_iv_).

In this study, we used E11.5 *Dbx1^nlsLacZ^* animals to genetically label the Dbx1 lineage of CR cells and carried out Reln/p73/βgal triple immunostaining and in situ hybridization for *Reln* and *p73* (Figure S1) in order to better define the distribution of the different CR populations in the pallial MZ. In rostral coronal sections (level L1, Figure 1A), βgal^+^/Reln^+^ cells were found all around the telencephalic vesicle and represented most of the Reln^+^ cells showing that they predominantly derived from the Dbx1 lineage [20]. In dorsomedial (DM) regions, nearly all βgal^+^/Reln^+^ cells also expressed p73 (Figure 1B) whereas in the dorsal (D) pallium 60% coexpressed p73 (unpublished data). Progressing from the dorsolateral (DL) to the lateral (L) pallium, the number of βgal^+^/Reln^+^ cells that did not coexpress p73 increased (Figure 1C). Less than 10% of hem-derived CR cells (Reln^+^/p73^+^/βgal^−^) were found on such rostral sections. Together with the lack of *p73* expression in the lateral pallium during earlier stages (Figure S1), these results show that *Dbx1*-derived CR cells originating from the septum express p73, whereas those from the PSB do not [26]. They appear to represent the main subpopulations in the medial and lateral pallium, respectively. At intermediate levels along the RC axis (level L2, Figure 1D), septum-derived (Reln^+^/p73^+^/βgal^+^) and hem-derived (Reln^+^/p73^+^/βgal^−^) CR cells represented, respectively, 60–70% and 20–30% of the total CR cells in DM regions (Figure 1E), whereas PSB-derived (Reln^+^/p73^−^/βgal^+^) represented the main CR cells population in L and DL territories (unpublished data and [20]). At caudal levels (level L3, Figure 1I), most dorsally located cells were derived from the hem (Figure 1J) whereas 50–60% of lateral cells originated within the PSB, as reported previously [20]. Co-labeling using βgal, Reln, and ER81, a protein expressed in rostral but not caudal CR cells at this stage [29], further confirmed the distribution of septum and PSB *Dbx1*-derived CR cells in the rostral pallium (unpublished data). Our results are consistent with a previous study showing that hem-derived CR neurons at E12.5 represent 90% and 60% of p73^+^ CR cells in the caudomedial and dorsolateral cortex, respectively [22].

Consistent with previous reports [26],[27], our data enable us to distinguish between hem-, PSB-, and septum-derived CR cells (respectively Reln^+^/p73^+^/βgal^−^, Reln^+^/p73^−^/βgal^+^, and Reln^+^/p73^+^/βgal^+^) and further confirm their ability to migrate over long distances from their generation site to become distributed in specific combinations in pallial territories (Figure 1K).

### Selective Ablation of Septum-Derived CR Cells in The Rostral Pallium

**Figure 2:**
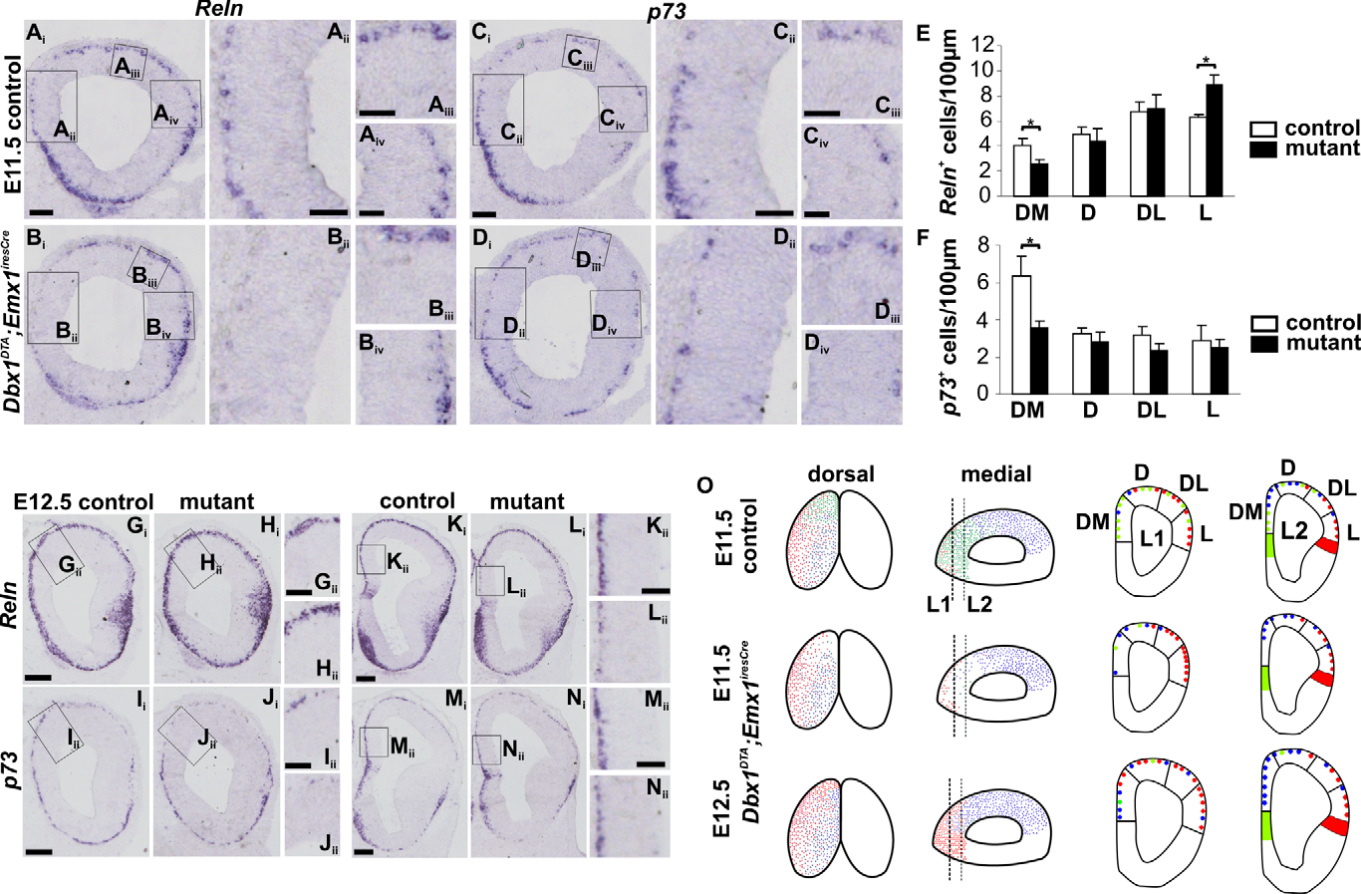
Loss of septum-derived CR neurons in the rostromedial preplate at E11.5 and compensation by hem- and PSB-derived CR cells at E12.5. (A–D) In situ hybridization with *Reln* (A,B) and *p73* (C,D) RNA probes at L1 levels of E11.5 control (A and C) and *Dbx1^DTA^;Emx1^iresCre^* (B and D) embryos. (A_ii_–A_iv_, B_ii_–B_iv_, C_ii_–C_iv_, and D_ii_–D_iv_) are high magnifications of boxed regions in (A_i_, B_i_, C_i_, and D_i_) and represent dorsomedial, dorsolateral and lateral territories, respectively. Quantifications of *Reln* (E) and *p73* (F) cell numbers per 100 µm. Histograms represent mean ± s.e.m. (*n* = 4). *P<0.05. (G–N) In situ hybridization with *Reln* (G, H, K, and L) and *p73* (I, J, M, and N) RNA probes at L1 (G–J) and L2 (K–N) levels on coronal sections of E12.5 control (G, I, K, and M) and *Dbx1^DTA^;Emx1^iresCre^* (H, J, L, and N) embryos. High magnifications of the DM region in L1 (boxes in G_i_, H_i_) show that *Reln* staining is similar between control (G_ii_) and mutant (H_ii_) embryos whereas less cells express *p73* in mutant embryos (compare [I_ii_] and [J_ii_]). On the contrary in DM regions at L2 levels, less cells express *Reln* in mutant embryos (compare [K_ii_] and [L_ii_]) whereas *p73* staining is comparable to controls (compare [M_ii_] and [N_ii_]). (O) Schematic representation of CR subtypes distribution in control and mutant embryos at E11.5 and E12.5. Septum-, hem-, and PSB-derived CR cells are represented in green, blue, and red, respectively. PSB-derived CR cells replace septum-derived CR cells lost upon ablation in L1 DM/D regions, whereas hem-derived CR cells populate L2 DM territories. Scale bars: 200 µm (G_i_, I_i_, K_i_, M_i_), 100 µm (A_i_, C_i_, G_ii_, I_ii_, K_ii_, M_ii_) and 50 µm (A_ii_–A_iv_, C_ii_–C_iv_).

To study the function of *Dbx1*-derived CR cells, we used *Dbx1^loxP-stop-loxP-DTA^* mouse mutants [20], which allow the conditional ablation of *Dbx1*-expressing cells in a temporally and spatially regulated manner, upon Cre-mediated recombination. Dbx1 is present in young postmitotic CR cells at L2 levels in the pallial (dorsal) septum, as shown by colabeling with Tuj1, Tbr1, and p73, whereas it is expressed in progenitors (Dbx1^+^/Tuj1^−^/Tbr1^−^) at the PSB (unpublished data and Figure 1F–H) starting at E10.5–E11.0 [20]. Dbx1 subpallial expression in the caudal septum/POA area is also detected at more caudal levels [20]. We used the *Emx1^iresCre^* line which shows effective recombination in pallial progenitors at E10.5, but recombines in a salt and pepper manner in the VZ of the PSB [22],[30]. On L2 sections of E11.5 *ROSA26^YFP^;Emx1^iresCre^* telencephalons, coexpression of YFP, and Dbx1 was detected in cells located in the MZ at the pallial septum, but not at the PSB (Figure S2A–B). We performed TUNEL staining at E11.5 and E12.5 to visualize the distribution of apoptotic cells (Figure S2C–D and unpublished data). At E11.5 in *Dbx1^DTA^;Emx1^iresCre^* embryos TUNEL^+^ cells were found in the septum at L2 levels but not in more caudal sections. Effective ablation was confirmed by the loss of Dbx1^+^ cells in the dorsal septum, but not in the ventral and caudal septum (Figure S2E–H and unpublished data). Some TUNEL^+^ cells were also detected in the mantle zone of the PSB. The identity of cells lost upon ablation was analyzed using *Reln* and *p73* in situ hybridization (Figure 2A–D, quantified in Figure 2E–F). At E11.5 in L1 sections, a strong reduction in the number of *Reln^+^* and *p73^+^* cells (a 40% and 45% loss, respectively) was detected in the DM pallium of mutant embryos when compared to controls (Figure 2A_ii_,B_ii_,C_ii_,D_ii_, E–F) in addition to a loss in the ventromedial region where septum-derived CR cells also migrate [20]. No decrease was detected in the DL, L and piriform cortices (Figure 2A_iii_,A_iv_,B_iii_,B_iv_,C_iii_,C_iv_,D_iii_,D_iv_). On the contrary, quantifications revealed an increase in the number of *Reln* ^+^ cells in the L pallium of E11.5 mutant embryos (Figure 2E), suggesting an increase in the production or the rostral migration of CR cells at the PSB. In caudal regions, where hem-derived CR cells constitute the main CR cells population, the numbers of *Reln^+^* and *p73^+^* cells were unchanged with respect to controls (Figure S2I–N). We conclude that *Dbx1^DTA^;Emx1^iresCre^* embryos present a specific partial loss of septum-derived CR cells in rostral DM territories of the developing pallium.

Interestingly, at E12.5, the number of *Reln^+^* cells in the rostral (L1) D and DM pallium of *Dbx1^DTA^;Emx1^iresCre^* embryos was similar to controls (Figure 2G–H and S2O). In contrast, that of *p73^+^* cells was still decreased (Figure 2I–J and S2O). At more caudal levels (L2), quantification of *p73^+^* cells showed no differences with respect to control embryos whereas *Reln^+^* cells were reduced in the DM pallium of mutant embryos (Figure 2K–N and S2P). These results indicate that CR cells from the PSB (Reln^+^/p73^−^) invade the mutant DM and D regions at rostral levels whereas young hem-derived CR cells (Reln^−^/p73^+^)[26],[27] migrate into DM regions at more caudal levels. This effect was already in progress at E12.0 (unpublished data). We conclude that a rapid compensatory migration of CR subtypes into the ablated rostral pallial territories occurs in less than one day following depletion of septum-derived CR cells (Figure 2O).

### Ablation of Septum-Derived CR Cells Leads to Early Regionalization Defects

The cortical primordium has been shown to be committed to a regional identity between E10.5 and E12.5 [8],[9]. The establishment of four opposing gradients of expression of TFs in response to signals from patterning centers at early stages (rostrolateral^high^ to mediocaudal^low^ for *Pax6*; rostromedial^high^ to caudolateral^low^ for *Sp8*; mediocaudal^high^ to rostrolateral^low^ for *Emx2* and caudolateral^high^ to mediorostral^low^ for *CoupTF1*) is crucial for the formation of tangential subdivisions of specialized cortical areas in the postnatal animal [2]–[5]. This corresponds to the time of generation of CR subtypes (E10.5–E11.5) [12],[13],[20] and indeed all three sources of CR subpopulations correspond to major signaling centers in the developing pallium (hem, septum, PSB). Furthermore, CR subtypes invade specific pallial territories between E10.5 and E12.5 and their distribution (Figure 2O) strongly correlates with the gradients of expression of TFs in the neuroepithelium. Specifically the distribution of hem-, PSB-, and septum-derived CR cells resembles the gradients of expression of *Emx2*, *Pax6*, and *Sp8*, respectively. These observations prompted us to analyze whether early cortical patterning and the establishment of gradients of *Pax6, Emx2, CoupTF1*, and *Sp8* expression were defective in the neuroepithelium of mutant animals at E11.5 and E12.5.

**Figure 3:**
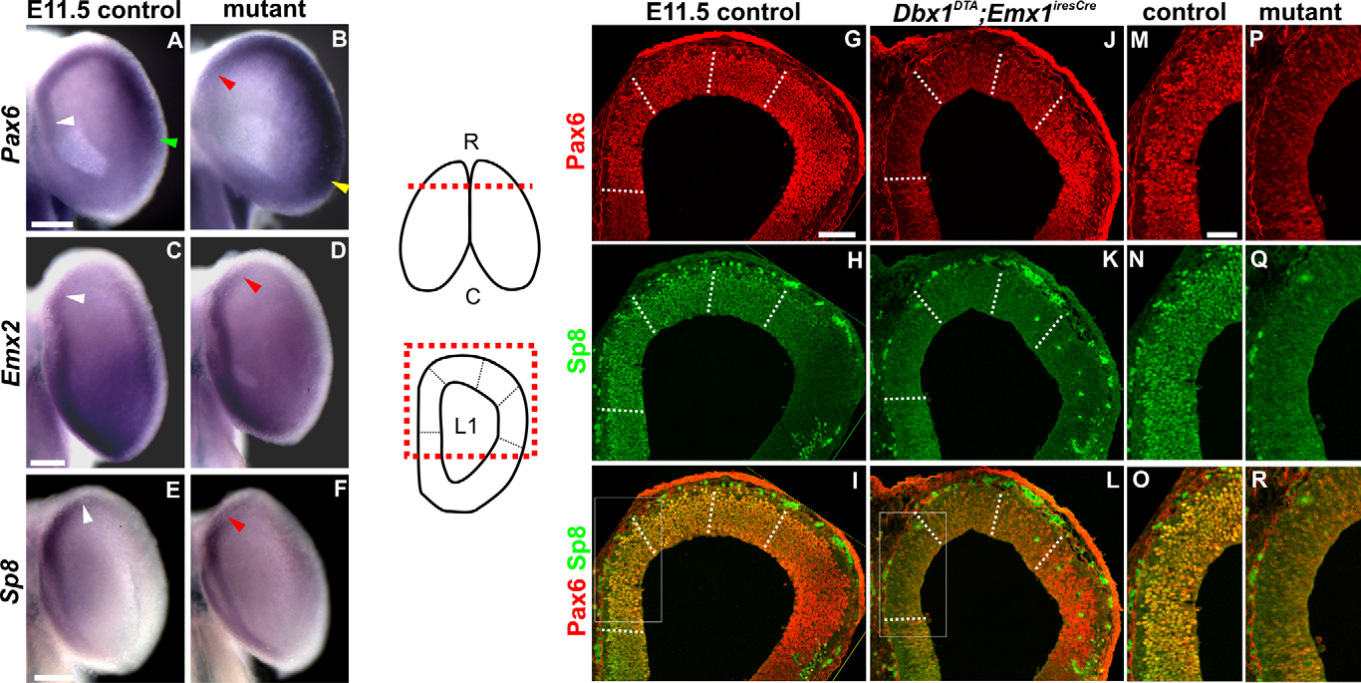
Ablation of septum *Dbx1*-derived CR cells leads to early regionalization defects. Whole mount in situ hybridization was carried out on E11.5 control (A, C, and E) and *Dbx1^DTA^;Emx1^iresCre^* mutant (B, D, and F) embryos using *Pax6* (A and B), *Emx2* (C and D) and *Sp8* (E and F) RNA probes (*n* = 3). In mutant embryos (B), *Pax6* expression is decreased in rostromedial regions compared to controls (compare white and red arrowheads in [A] and [B]) and increased in lateral regions (compare green and yellow arrowheads in [A] and [B]). On the contrary, the strong *Emx2* expression domain in medial regions is expanded rostrally in mutant embryos (D) compared to controls (C). In *Dbx1^DTA^;Emx1^iresCre^* embryos, *Sp8* expression is decreased in the most rostromedial territories (F) compared to controls (E). White and red arrowheads represent limits of expression domains in controls and in mutant brains, respectively. Coronal sections of E11.5 control (G–I and M–O) and *Dbx1^DTA^;Emx1^iresCre^* (J–L and P–R) embryos were immunostained with Pax6 (G, I, J, L, M, O, P, and R) and Sp8 (H, I, K, L, N, O, Q, and R). Left to this panel is represented a dorsal view of E11.5 brains (at the top). The red dashed line indicates the RC level (L1) of sections shown in (G–R). At the bottom, the red dashed box on a schematic coronal L1 section corresponds to images in (G,J,H,K,I,L). The dashed white lines define the borders of DM, D and DL regions as in Figures 1 and 2. (M,P,N,Q,O,R) are high magnifications of DM regions (white boxes in [I] and [L]). R, rostral; C, caudal. Scale bars: 1 mm (A–F), 100 µm (G–L) and 50 µm (M–R).

**Figure 4:**
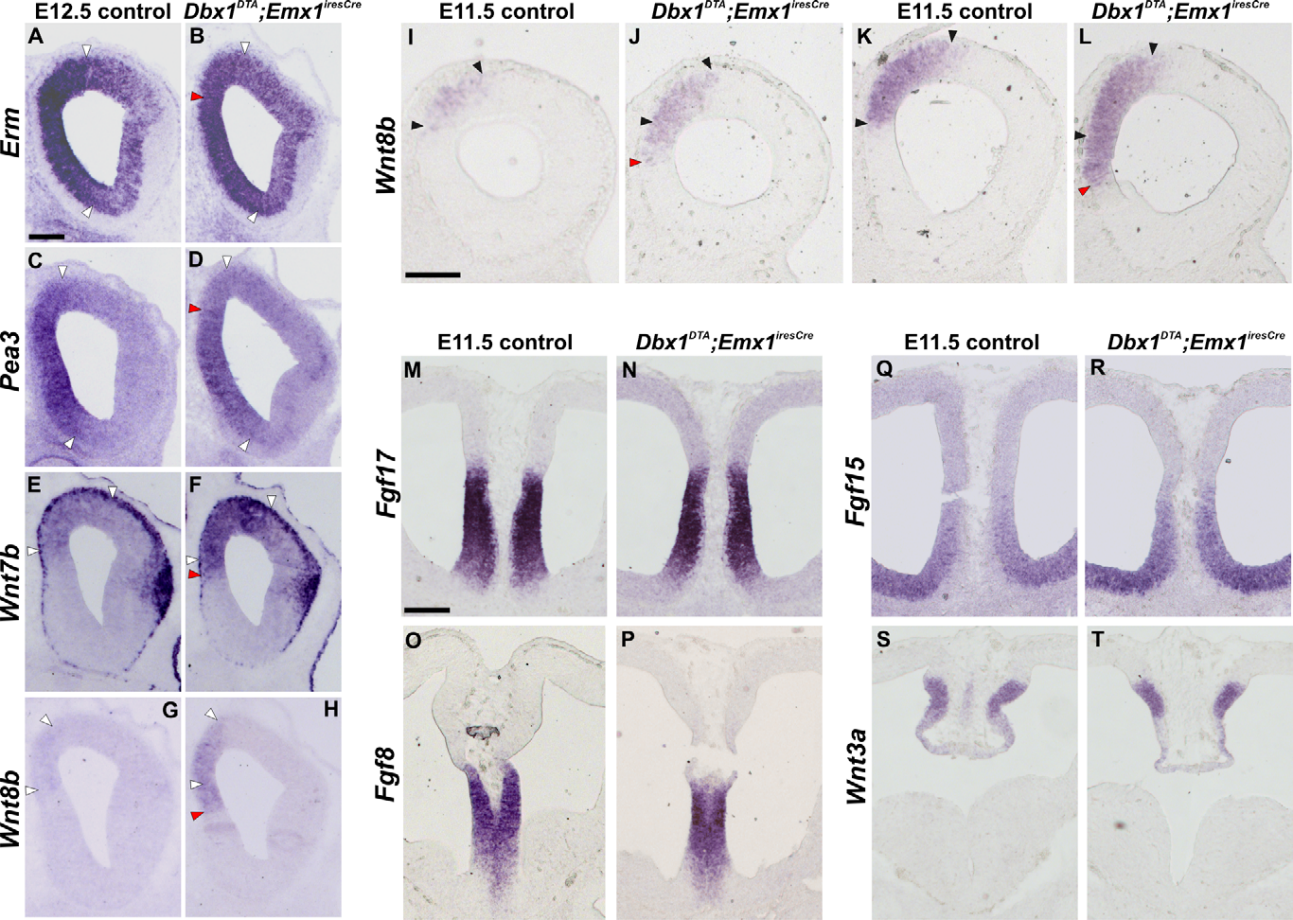
Changes in the regionalized expression of downstream effectors of Fgfs and of Wnts molecules in the absence of defects at signaling centers. (A–H) E12.5 coronal sections of control (A, C, E, and G) and *Dbx1^DTA^;Emx1^iresCre^* (B, D, F, H) telencephalons were hybridized with *Erm* (A and B), *Pea3* (C and D), *Wnt7b* (E and F), *Wnt8b* (G and H) RNA probes (*n* = 3). In mutant animals, *Erm* expression is reduced in DM/D and unaltered in ventromedial regions (compare [A] and [B]). *Pea3* expression in the DM/D domain is decreased in mutant embryos (compare [C] and [D]). W*nt7b* expression domain is extended ventrally in the medial pallium and its expression intensity is increased in mutant embryos (compare [E] and [F]). An increase in the size and intensity of the *Wnt8b* expression domain is detected in mutant animals in DM region (H) compared to controls in which, at this level, *Wnt8b* is barely detectable (G). White and red arrowheads indicate limits of high expression domains in controls and mutant animals, respectively. (I–L) In situ hybridization with *Wnt8b* RNA probe on E11.5 control (I and K) and *Dbx1^DTA^;Emx1^iresCre^* (J and L) embryos on two serial rostral coronal sections. The *Wnt8b* expression domain is expanded rostrally and ventrally in *Dbx1^DTA^;Emx1^iresCre^* embryos (J and L), compared to control (I and K) embryos. Black arrowheads delimit the *Wnt8b* expression domain in control, and red arrowheads the ventral limit of shifted domains in mutant embryos (*n* = 3). (M–T) In situ hybridization on E11.5 sections of control (M, O, Q, and S) and *Dbx1^DTA^;Emx1^iresCre^* embryos (N, P, R, and T) (*n* = 4). No difference is observed between control and mutant embryos for the expression of *Fgf17* (M and N), *Fgf8* (O and P), *Fgf15* (Q and R) and *Wnt3a* (S and T). Scale bars: 200 µm (A–T).

Initial analysis of *Pax6* expression in E11.5 control embryos using whole mounts in situ hybridization, showed this was low in the medial and high in the lateral pallium, respectively. In *Dbx1^DTA^;Emx1^iresCre^* embryos, *Pax6* expression was reduced in rostromedial and increased in lateral territories when compared to control embryos (Figure 3A,B). This reduction was confirmed using immunohistochemical localization of Pax6 protein at rostral L1 levels (Figure 3G,I,J,L,M,O,P,R) that also showed a decrease in D regions. Notably, by E12.5 an increase of Pax6 expression was observed in both rostromedial (DM) and dorsal (D) regions (Figure S3K,M,N,P) in mutant embryos, suggesting a lateral to medial shift in its expression following an initial downregulation within the medial and dorsal pallium. In control embryos, *Emx2* is expressed according to a caudal^high^ to rostral^low^ gradient at medial levels. In contrast, mutant embryos showed a rostral expansion in *Emx2* expression both at E11.5 and E12.5, as shown by in situ hybridization (Figure 3C,D and S3A–H), suggesting a caudal to rostral shift in its expression. *Sp8* is expressed with a rostromedial^high^ to caudolateral^low^ gradient in controls. In E11.5 mutant embryos, *Sp8* expression was found to be downregulated in the most rostromedial and dorsal pallium using whole mounts in situ hybridization as well as immunohistochemistry (Figure 3E,F,H,I,KL,N,O,Q,R). At E12.5 Sp8 expression was strongly upregulated in rostral D pallial territories (Figure S3L,M,O,P) and this extended to DL regions. *CoupTF1* is expressed at low levels in the rostromedial and high in the caudolateral pallium. Interestingly, the rostral domain of low *CoupTF1* expression appeared larger in mutant embryos at E12.5, but not at E11.5, (Figure S3I,J and unpublished data), suggesting a medial to lateral shift in its expression. Taken together, these results show that TFs expression gradients are displaced upon CR cells ablation and their redistribution. To further characterize the regionalization defects in septum CR cells depleted embryos, we analyzed four additional genes whose expression is also restricted to specific pallial territories, namely *Erm* and *Pea3* (involved in Fgfs signaling) and *Wnt7b* and *Wnt8b*. In E12.5 mutant embryos, *Erm* and *Pea3* expression was decreased in DM/D regions whereas that of *Wnt7b* in the VZ was increased compared to control embryos (Figure 4A–F). Interestingly, the expression of *Wnt7b* appeared decreased in the MZ. Remarkably, the *Wnt8b* expression domain in DM pallial regions was expanded rostrally and ventrally both at E11.5 and E12.5 (Figure 4G–L). These results strongly support the conclusion that regionalization is affected in mutant animals and show that *Wnt7b* and *Wnt8b* are strongly upregulated in cortical progenitors upon septum-CR cells ablation and redistribution.

Cortical regionalization is known to be controlled by secreted molecules expressed at signaling centers [2]–. However, expression of *Fgf8* and *Fgf17* in the septum/commissural plate and surrounding regions, of *Wnt3a* and *Wnt5a* at the hem, and of *Fgf15* and *Shh* in the subpallial rostral and caudal septum/preoptic area, respectively, were unaltered in *Dbx1^DTA^;Emx1^iresCre^* embryos along both the RC and DV axis between E11.0 and E12.5 (Figure 4M–T, Figure S4A–B and unpublished data). We also did not observe changes in the expression domains of *Msx1* and *BMP4* at the roof plate (Figure S4C–D and unpublished data). Furthermore, dorsoventral patterning at the septum was similar to controls, as shown by the position of the Ngn2 and Mash1 ventral and dorsal limits of expression (Figure S4G–N), respectively, and the dorsal limit of *Fgf15* (Figure 4Q–R). Lastly, no changes in the ventral limit of expression of *Gli3* were observed (Figure S4E–F) as well as in the expression of genes at the PSB (*Sfrp2* and *Tgfα*) were observed (unpublished data). Together, these results show that septum-derived CR cells loss at E11.5 and the subsequent compensation by hem- and PSB-derived CR subtypes affect regionalization of the rostromedial and dorsal neuroepithelium without altering gene expression at signaling centers. The downregulation of *Sp8*/*Pax6* at E11.5 and of *Erm*/*Pea3* at E12.5 in the dorsomedial pallium correlate with loss of septum-derived CR cells and suggests a persistent loss of Fgf signaling. At E12.5 the increase in *Emx2/Wnt7b/Wnt8b* expression in DM territories and the upregulation of *Sp8/Pax6* in DM and D regions correlate with compensation by hem- and PSB-derived CR cells.

### Defects in Proliferation of VZ Progenitors and Neurogenesis upon CR Cells Ablation

**Figure 5:**
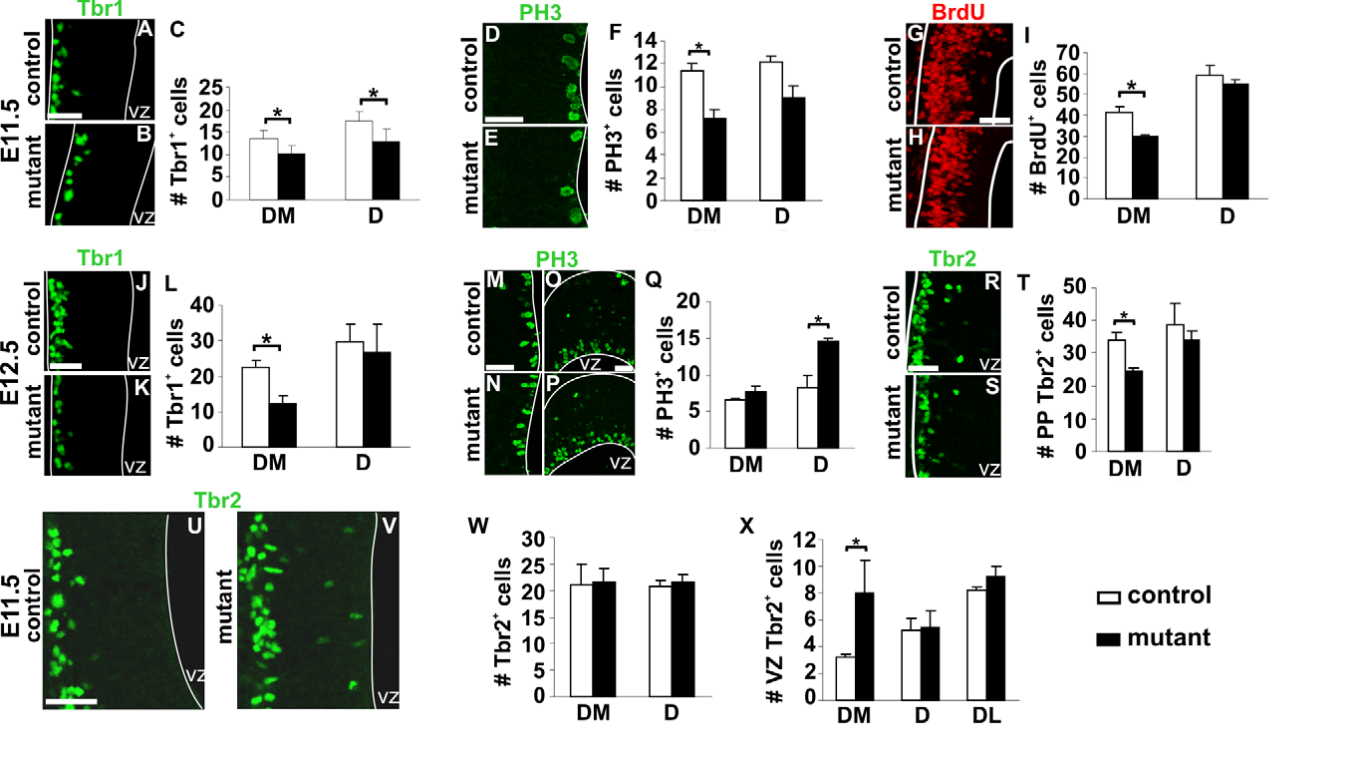
Temporary proliferation and neurogenesis defects upon CR cells ablation. Coronal sections of E11.5 control (A, D, G, and U) and *Dbx1^DTA^;Emx1^iresCre^* (B, E, H, and V) embryos immunostained with Tbr1 (A and B), PH3 (D and E), BrdU (G and H) and Tbr2 (U and V). For all quantifications, histograms represent numbers of cells per 100 µm (mean ± s.e.m) for controls (white) and mutant embryos (black). At E11.5, the numbers of Tbr1^+^, PH3^+^ and BrdU^+^ (one-hour pulse) cells are decreased in DM territories of mutant animals (C, F, I, *n* = 5 for C and F, *n* = 3 for I). However, preplate Tbr2^+^ cells number is not significantly different between control and mutant embryos (W, *n* = 5). Immunostaining with Tbr1 (J and K), PH3 (M-P) and Tbr2 (R and S) on sections of E12.5 control (J, M, O, and R) and *Dbx1^DTA^;Emx1^iresCre^* (K, N, P, and S) embryos. The number of Tbr1^+^ and Tbr2^+^ cells is decreased in DM region of mutant telencephalons (L, T, *n* = 5). The number of PH3^+^ cells is increased in the D (Q, *n* = 5) but not DM regions. (U, V) Increase in Tbr2^+^ cells located close to the ventricle in mutant embryos at E11.5 (X, *n* = 5). *P<0.05. Scale bars: 50 µm.

Changes in patterning of the early neuroepithelium prompted us to analyze neurogenesis in mutant animals. At E11.5, Tbr1 labels preplate postmitotic glutamatergic neurons, including CR cells [31]. In mutant embryos Tbr1^+^ cells were detected in the correct location, but we observed a 30% decrease in their number in DM and D territories of the rostral pallium (Figure 5A–C), corresponding to Tbr1^+^ septum-derived CR cells loss. At E12.5, when compensation by other CR cells subtypes had already occurred, Tbr1^+^ cell number was decreased to 50% in the DM pallium, but no changes were observed in other pallial regions (Figure 5J–L and unpublished data). Decrease of *Wnt7b* expression in the MZ was also observed at E12.5 correlating with reduced differentiation (Figure 4E,F). Similar numbers of Tbr1^+^ neurons in rostral DM regions of control and mutant embryos was observed by E13.0 (unpublished data). Quantifications of the number of mitotic cells using immunostaining for PH3 and BrdU following a one-hour pulse at E11.5 revealed that proliferation was decreased in the region depleted in CR cells (Figure 5D–I). At E12.5, the number of mitotic cells in DM was similar in control and mutant embryos, indicating that the number of VZ progenitors undergoing mitosis is decreased temporarily at E11.5 (Figure 5M,N,Q). We also detected an increased number of PH3^+^ cells at E12.5 in D regions, where Reln^+^ cells from the PSB had repopulated the preplate (Figure 5O–Q). Thus, septum-derived CR cells loss results in a transient decrease of proliferation at E11.5 in the DM pallium. Repopulation of D regions by PSB-derived CR subtypes correlates with an increase in mitosis of cortical progenitors.

To further dissect the properties of the early neuroepithelium upon depletion of CR cells in the preplate, we analyzed the expression of Tbr2, a marker of early postmitotic cells in the MZ and of basal/intermediate progenitors [32]. Tbr2^+^ cell number in the preplate at E11.5 was similar in control and mutant embryos (Figure 5W), but decreased at E12.5 in DM regions in mutant embryos (Figure 5R–T). Notably, at E11.5, but not at E12.5, we observed Tbr2^+^ cells ectopically positioned at the apical side of the neuroepithelium flanking the ventricle in the mutant DM region (Figure 5U,V,X). Together, these studies show that the distribution of CR subtypes in the MZ influences the proliferation and differentiation of progenitor cells within the VZ.

### The Position of Cortical Areas Is Shifted upon Septum CR Cells Ablation

**Figure 6:**
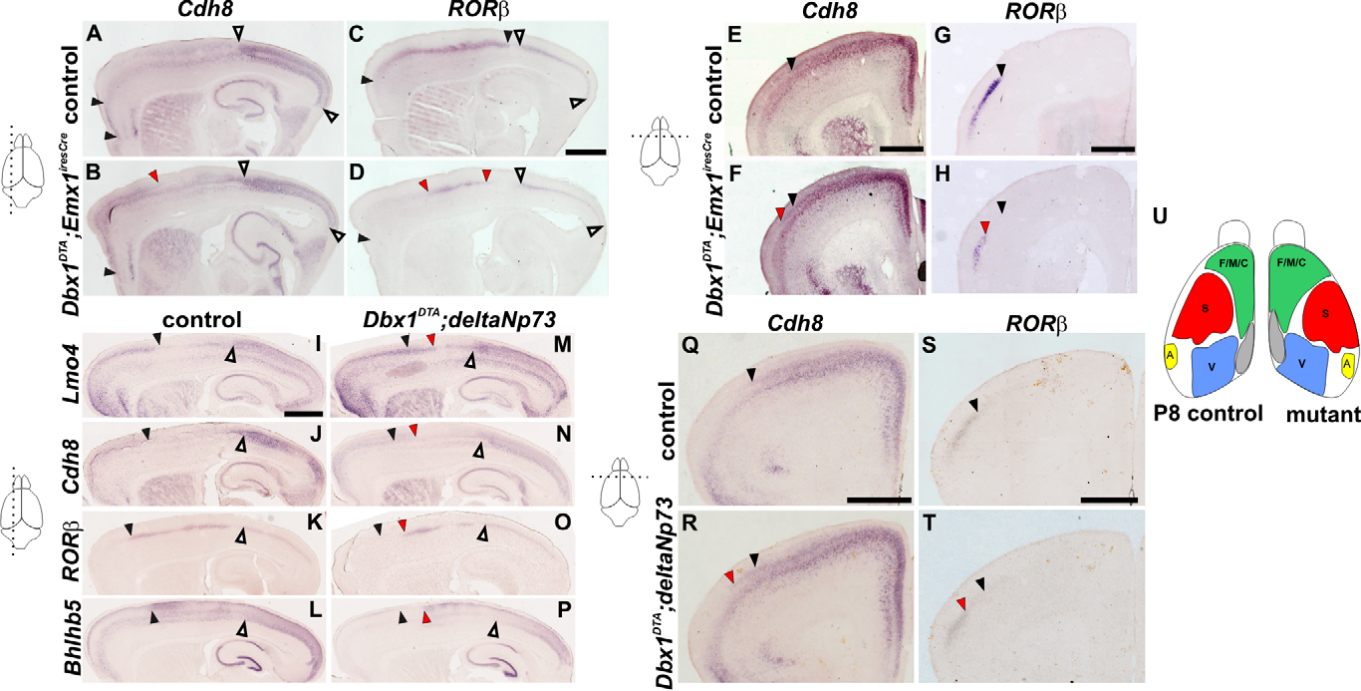
Shifts in position of cortical territories upon CR cells ablation. (A–H) In situ hybridization with *Cdh8* (A, B, E, and F) and *RORβ* (C, D, G, and H) RNA probes on sagittal (A–D) and coronal (E–H) sections of P8 control (A, C, E, and G) and *Dbx1^DTA^;Emx1^iresCre^* (B, D, F, and H) brains. On sagittal sections at lateral level (A–D) the rostral *Cdh8* expression domain is extended caudally in mutant brains (B), whereas the caudal domain is similar between controls (A) and mutant animals (B). The rostral *RORβ* expression domain is reduced but the caudal visual domain does not change in mutant brains (D) compared to controls (C). Black and white arrowheads represent rostral and caudal expression domains, respectively, in controls and red arrowheads indicate the displacement of boundaries of expression in mutant brains. On coronal sections at rostral levels, the *RORβ* expression domain is reduced laterally in mutant brains (H) compared to controls (G), corresponding to a medial to lateral expansion of the *Cdh8* expression domain (compare [E] and [F]). Sagittal sections of P8 control and *Dbx1^DTA^;deltaNp73* brains were hybridized with *Lmo4* (I and M), *Cdh8* (J and N), *RORβ* (K and O) and *Bhlhb5* (L and P) RNA probes. The rostral domain corresponding to superficial expression of *Lmo4* and *Cdh8* is enlarged caudally in mutant animals (M–N) compared to controls (I–J), correlating with a reduction of the position of the somatosensory area, as visualized by *RORβ* and *Bhlhb5* boundaries (K, O and L, P). Black and white arrowheads represent rostral and caudal expression domains, respectively, in controls and red arrowheads show the displacement of boundaries in mutant brains. Coronal sections of *Dbx1^DTA^;deltaNp73* P8 brains hybridized with *Cdh8* (Q and R) and *RORβ* (S and T) show similar defects as in *Dbx1^DTA^;Emx1^iresCre^* brains. (U) Summary of the changes in position and size of the different cortical areas, on a dorsal view of P8 control and *Dbx1^DTA^;Emx1^iresCre^* and *Dbx1^DTA^;deltaNp73* (mutant) brains. F/M/C, frontal/motor/cingular; S, somatosensory; V, visual; A, auditory. Scale bars: 1 mm.

Changes in TFs gradients prompted us to investigate whether arealization was affected in ablated animals. In control brains, *Cdh8* is highly expressed in layers II/III of the frontal/motor cortex and visual areas [33],[34]. *RORβ* is restricted to layer IV in the somatosensory and visual areas [35]. On sagittal sections of P8 control brains at lateral levels, *RORβ* was expressed in the most rostral territories, where *Cdh8* expression in superficial layers was not detected (Figure 6A,C). At corresponding lateral levels in *Dbx1^DTA^;Emx1^iresCre^* mutant brains a large domain of *Cdh8* expression in superficial layers was observed in rostral territories (Figure 6A–B). Serial sections hybridized with *RORβ* showed a lack of expression in the rostral region expressing *Cdh8* (Figure 6C,D) indicating that, at this level, somatosensory area is replaced by frontal/motor area. The size of the caudal domain of *Cdh8* expression, corresponding to the visual area, appeared normal at this level. Notably, a small caudal domain, where *RORβ* is normally absent in control brains, likely corresponding to the lateral parietal association cortex, appeared enlarged in mutant brains (Figure 6C,D). Analysis of coronal sections at rostral levels showed a lateral shift of motor areas at the expense of somatosensory areas (Figure 6E–H). However, at more caudal levels the size of the *RORβ* expression domain appeared normal. *RORβ* expression analysis together with SERT immunostaining which identifies the barrelfield showed that primary somatosensory territories did not appear to change size or identity, but rather that their positioning was displaced caudally and laterally (unpublished data). Sagittal sections at medial levels also showed that the rostral domain of *Cdh8* expression was reduced, whereas the caudal domain, corresponding to retrosplenial cortex, was rostrally displaced in mutant animals (Figure S5S,T). Lastly, the characteristic high expression of *Cdh8* within lower layers of retrosplenial territories appeared to be shifted more rostrally, at the expense of cingulum regions (Figure S5S,T).

Quantifications of relative cortical area sizes on whole mount P0 brains, using *Cdh8* (Figure S5A–D) and *Lmo4* (unpublished data) as markers, showed a small but significant increase in the frontal/motor cortex with no differences in visual regions or overall neocortical size. Finally, although CR cells have been shown to play a role in cortical layers formation, lamination in each area appeared to be fairly normal (Figure 6 and Figure S5I–N), as reported upon hem-derived CR cells ablation [22].

Together, these results show that deletion of septum CR cells results in the rostral displacement of the retrosplenial cortex, an increase in the size of motor area and a medial-to-lateral and rostral-to-caudal shift in the position of the somatosensory area (Figure 6U). To further confirm that the observed defects were due to CR cells ablation, we used the *deltaNp73* mouse line, which specifically labels and targets Cre recombination in CR subtypes [24]. *Dbx1^DTA^;deltaNp73* embryos at E11.5 showed a specific decrease of septum-derived CR neurons (*Reln*, *p73*, TUNEL staining, Figure S5O–R and unpublished data) together with changes in cortical regionalization (*Pax6*, *Sp8*, *Emx2*), similar to ablation using *Emx1^iresCre^* animals (unpublished data). Analysis of *Cdh8*, *RORβ* and *Lmo4* expression in P0 (Figure S5E–H and unpublished data) and P8 *Dbx1^DTA^;deltaNp73* animals using *Lmo4, Cdh8*, *RORβ*, and *Bhlhb5* confirmed an increase in the size of motor area and a caudolateral shift in the positioning of the somatosensory area (Figure 6I–T). Thus, we conclude that CR neurons mediate the regionalization/arealization defects observed in mutant animals.

### FACS-Sorted Septum *Dbx1*-Derived CR Cells Express Multiple Secreted Molecules

To gain insights into the mechanisms by which CR neurons affect cortical regionalization, we used microarray analysis of purified septum *Dbx1*-derived CR cells to identify candidate secreted signaling molecules. Dorsomedial (DM) and dorsolateral (DL) pallial regions at rostral L1/L2 levels were dissected from E12.5 *Dbx1^CRE^;ROSA26^YFP^* embryos. Our data showed that most YFP^+^ cells in the DM part (≥95%) were Reln^+^/p73^+^ septum-derived CR cells whereas the DL one was enriched in Reln^+^ PSB-derived CR cells (∼55–65%) (see Materials and Methods, Figure S6A–C, [20] and unpublished data). YFP^+^ *Dbx1*-derived cells were FACS-sorted (Figure S6D–I) and RNA expression profile was analyzed using Affymetrix whole mouse transcript microarrays.

**Figure 7:**
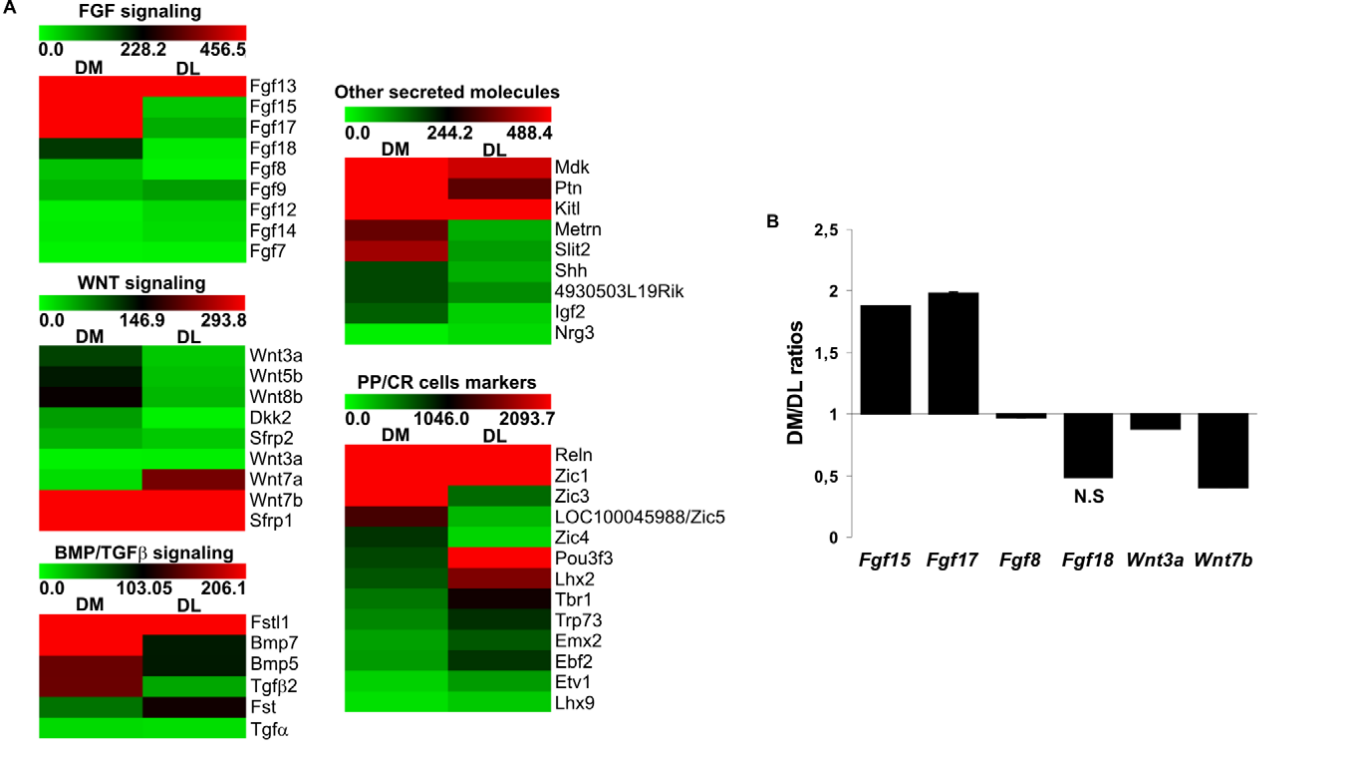
Heatmap representation of transcript-expression profiles of FACS-sorted *Dbx1*-derived cells and validation by qPCR. (A) This dataset includes the ProbeSets with significant differential expression (*P*<0.0005) (except for *Fgf7*, *Tgfα, Shh* and *Wnt3a* (*P*>0.5)), grouped in Fgf, Wnt, Bmp/Tgfβ signaling pathways, other secreted molecules and preplate/CR cells markers. Each row denotes a single gene and left and right columns represent expression in YFP^+^ cells purified from DM and DL pallial regions, respectively. In this false-color image, increasing red intensities denote genes with high expression levels and increasing green intensities genes with low expression values (see scale bars above each expression set). The raw Affymetrix expression values and *p* values for each ProbeSet are listed in Table S1. The *Trp73* ProbeSet found to be expressed in the DM and DL samples corresponds to the full-length transcript and does not allow for the discrimination between the multiple isoforms for this gene. (B) Histograms represent the expression value ratio (triplicate mean ± s.d.) of different Fgfs and Wnts enriched in YFP^+^ cells purified from DM with respect to DL pallial regions. The values of the ratios are listed in Table S2. The ratios are statistically significant (P<0.05) except for Fgf18. N.S: not significant. The expression levels of the different genes in DM and DL, normalized to the reference gene rpS17, are listed in Table S2.

The identity of purified CR cells was confirmed by the presence of Reln and p73 in both DM and DL sorted cells together with the expression of other preplate markers (Figure 7A). We found that multiple secreted molecules were differentially expressed in DM and DL YFP^+^ cells (Figure 7A). Notably, we detected ∼10-fold higher levels of *Fgf15*, *Fgf17* and *Fgf18* in DM with respect to DL cells (Figure 7A and Table S1). In particular, *Fgf15* and *Fgf17* were expressed at high levels in the DM population, which contrasted with their relatively low levels of expression of *Fgf18* and *Fgf8* (Figure 7A and Table S1). In addition, although the total detected levels of expression were quite low, the expression of several other genes that encode secreted signaling factors (*Wnt5a*, *Wnt5b*, *Wnt8b*, *Tgfβ2*, *Dkk2*, *Fstl1*, *Slit2,* and *Igf2)* was comparatively ≥3–5 fold higher in DM CR cells (Figure 7A and Table S1). Further analysis of the expression of six genes (*Fgf15, Fgf17, Fgf18, Fgf8, Wnt3a*, and *Wnt7b*) using qPCR in YFP^+^ sorted DM and DL cells confirmed that *Fgf15* and *Fgf17* were expressed at higher levels in DM versus DL cells (Figure 7B and Table S2). The expression of *Fgf18* was more variable and did not show a significant difference between the two cell types, likely reflecting the low levels of expression revealed by microarray analysis. qPCR analysis also confirmed the absence of *Fgf8* and *Wnt3a* in both DM and DL cells and a higher expression of *Wnt7b* in DL cells.

These results show that septum-derived CR neurons express a highly specific repertoire of signaling factors and, in particular, high levels of *Fgf15* and *Fgf17*, which have well-established roles in cortical patterning. Together, our data suggest that secretion of signaling molecules might be one of the mechanisms by which CR neurons contribute to cortical patterning, thereby enabling a refined interplay of multiple signaling pathways which might be crucial to this end.

## Discussion

In this study, we show that specific combinations of CR subtypes dynamically populate distinct regions of the developing pallium. Genetic ablation of septum *Dbx1*-derived CR cells and redistribution of PSB- and hem-derived CR subtypes between E11.0 and E12.5 in cortical territories result in changes in early patterning events and progenitor cell division and differentiation at long distance from CR cells generation sites. These early regionalization defects correlate with changes in the size and positioning of cortical areas at postnatal stages, without affecting signaling centers. Moreover, we show that septum-derived CR cells express a specific combination of secreted factors. Together, our results show that the distribution of CR subtypes in the preplate/MZ controls VZ progenitor properties and strongly point to a novel role of CR cells subtypes as mediators of early cortical patterning.

### Dynamic Distribution of CR Cells Subtypes in the Early Preplate and Their Redistribution upon Ablation

The molecular identity of CR neurons has been subject to debate and three main markers have been considered to be expressed in CR cells: Reln, p73, and Calretinin [12],[13],[20],[26],[27]. Together with previous reports [26],[27], our data show that p73 is expressed in CR cells generated at the hem- and the septum- but not in PSB-derived CR neurons. Reln appears to be expressed by all CR subtypes although the onset of its expression might be slightly delayed in hem- and septum-, with respect to PSB-derived cells. By studying the time course of the expression of these markers, we were able to map the distribution of the distinct subtypes and to show that upon elimination of septum *Dbx1*-derived CR cells, rostral dorsal territories are repopulated by PSB-derived CR cells and medial regions by hem-derived CR cells. The dynamic redistribution of CR subtypes upon ablation occurs very rapidly within a 24-hour period and is mostly accomplished by E12.5. Our results strongly point to the existence of a crosstalk between CR cells involved in regulating invasion of cortical territories at early stages of development, which are in agreement with previous reports suggesting that contact-inhibitory interactions between CR cells might control their dispersion throughout the surface of the cortex [20],[36]. Our data suggest that the sites of generation, the birthdates and the onset/speed of migration of CR subtypes are crucial for their kinetics of arrival and, thus, their distribution in pallial regions, and that these form a precise molecular map at E12.5. Small variations of these parameters might therefore have profound consequences on the construction of this map and may possibly occur among individuals. Invasion of neocortical territories by CR cells derived from other sources has also been shown in hem-ablated mouse mutants [22]. However, the suggested progressive increase of hem-derived CR cells in prospective neocortical regions at later stages of development, together with an almost complete loss of CR neurons in the neocortex of these mutants, might indicate that the distribution of CR neurons from midcorticogenesis is controlled by additional mechanisms, including selective survival of CR subtypes.

### CR Cells Loss Affects the Relative Size and Positioning Of Prospective Cortical Territories

Multiple signaling centers or “organizers” have been shown to be involved in the induction and patterning of early telencephalic territories. In the cortical primordium, signaling molecules are thought to control the graded expression of TFs, among which *Emx2*, *Pax6*, *CoupTF1*, and *Sp8* are involved in early cortical regionalization and arealization [2]–[5],[37]. We have shown that loss of CR cells correlates with opposite changes in *Sp8*/*Pax6* and *Emx2/Wnt8b* expression in DM/D pallium at E11.5, whereas compensation by hem- and PSB-derived CR cells correlates with those in *Emx2/Wnt7b/Wnt8b* and *Pax6*/*CoupTF1* at E12.5, respectively. These early regionalization changes parallel defects observed in arealization in postnatal animals. These results *s*trongly support the notion that a fine regulation of the levels of expression of TFs, as well as that of players in the Wnt and Fgf signaling cascades, in each territory controls cortical areas positioning and size according to the “cooperative concentration model” [38]. Our data suggest that the regions of intersection of regionalization gradients are highly sensitive to changes and are crucial for setting up the borders of cortical areas. To complement “loss-of-function” (ablation of septum CR cells) and “gain-of-function” (repopulation by other CR subtypes) in vivo, we have attempted to perform grafting experiments in vitro using FACS sorted septum-derived CR cells. However, technical limitations, related to the number of sorted cells together with the culture conditions which do not preserve regionalization gradients of TFs or neurogenesis as in vivo, render such paradigm extremely challenging, if possible.

Our results are consistent with loss and gain-of-function experiments in mice. An anterior expansion of *Wnt8b* expression occurs upon a decrease in Fgf8 signaling [39] as well as in *Pax6* mutants [40], whereas a reduction is detected in *Emx2* mutants [40]. Defects of frontal cortical regions and a caudalization of medial cortical territories when septum-derived CR cells are ablated recapitulate some of the defects observed in *Fgf17* and *Fgf8* mutants and is consistent with an antagonistic regulation of *Erm*, *Pea3*, and *Sp8* by Fgfs and Emx2/Wnt signaling [41]. Since *Sp8*, *Erm*, and *Pea3* are genes induced by Fgf signaling [39],[42],[43] and Wnts are involved in graded expression of *Emx2* [44], our results are consistent with septum CR cells being involved in maintaining *Pea3* and *Sp8* and, thus, mediating Fgf signaling. The expression of multiple Fgfs in purified septum-derived CR cells also parallels the changes in the regional-specific expression of downstream targets of the Fgf cascade in mutant animals. Furthermore, an expanded *Pax6* expression at E12.5 at rostral dorsal levels correlates with an increase in motor area size and, together with a decrease in *CoupTF1* expression, in a shift in the positioning of motor and somatosensory areas. Conversely, a loss of *Dbx1* expression at the PSB, as observed in *NesCre;Dbx1^DTA^* (unpublished data) or *Pax6* mutants [45], correlates with a decrease in the size of the motor cortex in these mutants. Together, these data suggest that PSB-derived CR cells are involved in mediating anti-hem signaling.

In addition, except for few studies in which Reln or Calretinin have been globally analyzed, such as in *Emx2*, *Pax6*, and *CoupTF1* mutants [46]–[48], CR subtypes have not been analyzed in detail or at early enough stages leaving open the possibility that some of the effects observed in mutants for genes involved in regionalization/arealization might be mediated by differences in CR cells generation and/or their migration. Moreover, in *p73* mutants differences in Calretinin expression at early stages were correlated with a dorsal shift of the entorhinal cortex and a reduced size of the occipital and posterior temporal areas [49], suggesting an involvement of CR cells in cortical arealization.

All three sites of CR subtypes generation coincide with patterning centers. *Dbx1* expression domains at the septum and the PSB both reside in the immediate vicinity of, and possibly overlap with, regions highly enriched in Fgf and Wnt antagonists signaling. Inasmuch as no differences were observed in the domain of expression of *Fgf8*, *Fgf17, Fgf15, Wnt3a, Msx1*, and *Shh* as well as in DV patterning, it seems unlikely that CR cells ablation affects signaling centers themselves, and we rather propose that it modulates steps downstream from them. This is consistent with the timing of birth and ablation of CR cells, which occur at E10.5–E11.0, and, thus, later than the period when gene expression at signaling centers is not yet fixed, as in the case of the increase of *Fgf8* in *Emx2* mutants at E9.0 [42]. Moreover, it was recently demonstrated, using gain-of-function experiments, that *Fgf8* induces the generation of rostral CR subtypes and that *Fgf8*, *Pax6* and *Emx2* loss-of-function mutants present defects in CR subtypes specification [29]. In the present manuscript we show that additional sources of Fgf15 and Fgf17 from migrating septum-derived CR cells exist in the developing pallium. Fgf8 has been shown to lie upstream of other Fgfs, notably Fgf17, and Fgf15 to be a modulator of Fgf8 signaling [41],[50]. Together, these data suggest that the role of Fgf8 in cortical regionalization and arealization might be mediated, in addition to passive diffusion, by secretion of morphogens by migrating Cajal-Retzius neurons. A fine tuning of the concentration of these factors is likely to tightly balance proliferation and differentiation of cortical progenitors. These results also unravel a mechanism by which Fgf8 might affect regionalization, which up to now has possibly been underestimated.

### Role of CR Subtypes in Cell Division of Cortical Progenitors and Neurogenesis

A rostro-lateral^high^ to caudo-medial^low^ gradient of neurogenesis has been shown to distinguish pallial territories during development and to correlate with differences in neuronal numbers and lamination in cortical areas [51],[52]. How the molecular mechanisms controlling neurogenesis and lamination are linked to those of regionalization and arealization is still an open question. We show that septum-derived CR cells loss at E11.5 results in a decrease in proliferation (PH3^+^) and an increase of apical Tbr2^+^ cells, suggesting the precocious generation of postmitotic preplate neurons (Tuj1^+^, unpublished data) at the expenses of neuroepithelial self-renewing cell divisions [53]. By E12.5, a recovery in rostral DM proliferation and an increase in mitosis in D regions correlate with compensation by hem-derived and PSB-derived CR cells, respectively. This transient and early effect (even before the generation of layer VI neurons) is consistent with no major differences in the number of neurons in deep cortical layers. Since lower frequencies of differentiative divisions have been reported during early corticogenesis in *reeler* mutants [54] and we detect no differences in Tbr2 staining in these mutants (unpublished data), it is unlikely that Reln is responsible of the effects observed at E11.5. We find that ablation of the septum CR subtype does also not result in “inverted” cortical lamination, as would be expected from a loss in Reln signaling as in the *reeler* mutants. This is consistent with the absence of defects in layers formation in the neocortex of hem-ablated mutants [22] and strongly suggests that low Reln levels are sufficient to preserve cortical lamination [55]. Furthermore, defects in arealization have not been reported in *reeler* mutants [54], although the expression of molecular markers has not been so far analyzed in these mutants. Together these results strongly suggest that since all CR cells subtypes express Reln, and that hem- and PSB-derived CR cells rapidly repopulate the ablated regions, the role of CR cells in cortical arealization appears to be Reln-independent.

### Long-Distance Patterning: CR Cells as “Migrating” Signaling Units?

Passive diffusion occurs very efficiently at short time scales over a few dozens of cell diameters [56]. Various mechanisms can influence morphogen delivery in tissues surrounding a source, such as endocytosis and subsequent degradation [57], trapping in the extracellular matrix [58] or diffusion in the ventricular fluid [59],[60]. Nevertheless, as development proceeds and the cortex grows, this results in the increase in the distance between signaling centers. Additional mechanisms might be necessary to maintain and coordinate the growth and spatial patterning of the cortex and, thus, the robustness of morphogens signaling. Examples of “migrating” cells/structures affecting the development of distant territories are neural crest cells originating at the mid-forebrain junction and affecting craniofacial and anterior neural tube growth/survival in chick [61]–[63], and axonal projections affecting cell cycle progression of cortical progenitors [64]. CR cells are generated in regions highly enriched in signaling molecules and migrate in close contact with the early cerebral cortex neuroepithelium. Co-culture experiments with semipermeable membranes in the cerebellum provided evidence that neocortical CR cells release soluble signals other than Reln, thereby influencing the radial glia phenotype [18]. Even though we do not rule out additional mechanisms, such as CR cells carrying patterning molecules tethered to their cell surface or forming cell-cell contacts with radial glia basal attachments, our data strongly suggest that different CR subtypes act as mediators of cortical patterning by secreting a variety of ligands, including Fgfs and Wnts. Our data hint at CR cells participating in the fine tuning of multiple signaling pathways which is likely to underlie the regulation of cortical regionalization. Thus, we propose that these highly motile cells have a crucial role and serve as “mobile patterning units” at early stages of development.

## Materials and Methods

### Ethics Statement

All animals were handled in strict accordance with good animal practice as defined by the relevant national and/or local animal welfare bodies, and all mouse work was approved by the Veterinary Services of Paris (Authorization number: 75-1454).

### Animals

In this study, we used a *Dbx1^nlsLacZ^* mouse line [20],[65] to trace *Dbx1*-derived cells. *Dbx1^nlsLacZ/+^* embryos allow the transiently labelling of *Dbx1*-derived cells starting at their generation site and during the first phases of their tangential migration, due to the persistence of the β-galactosidase protein in the cells. In order to analyze the effect of eliminating *Dbx1*-derived CR cells, we inserted an *IRES-loxP-stop-pGKneo-loxP-DTA* (diphtheria toxin) cassette into the *dbx1* locus by homologous recombination (*Dbx1^loxP-stop-loxP-DTA^*) [20]. A functional DTA is expressed exclusively upon Cre-mediated recombination. Mutant animals were crossed with a *Emx1^iresCre^* [30] mouse line which expresses the Cre recombinase in pallial progenitors. *Dbx1^loxP-stop-loxP-DTA^* and *Emx1^iresCre^* animals were used as controls for all experiments. *ROSA26^loxP-stop-loxP-YFP^* mice [66] crossed with *Emx1^iresCre^* and *Dbx1^CRE/+^* [20] were used to permanently trace cells derived from *Emx1*- and *Dbx1*-expressing cells, respectively. Permanent tracing using *Dbx1^CRE^;ROSA26^YFP^* embryos is very similar to that using *Dbx1^CRE^; βactin:lacZ* and *Dbx1^CRE^;TAU^GFP^* at E12.5, as previously reported [20]. We also used *deltaNp73* animals which have been engineered to express the Cre recombinase in the *p73* locus and, thus, in CR subpopulations [24]. Embryos and postnatal animals were genotyped by PCR using primers specific for the different alleles. For BrdU experiments, E11.5 embryos were obtained from females injected intraperitoneally with a single dose of BrdU (50 mg/kg) one hour prior to collection.

### Tissue Preparation, In Situ Hybridization, and Immunohistochemistry

For staging of embryos, midday of the vaginal plug was considered as embryonic day 0.5 (E0.5). Embryos for immunohistochemistry were fixed by immersion in 4% PFA, 0.1 M phosphate buffer (PB) pH 7.2 for 2 h at 4°C and rinsed in PBS for 2 h. Postnatal animals were anesthetized and perfused with 4% PFA for 10 min. Brains were cryoprotected overnight in 30% sucrose, 0.1 M PB and embedded in O.C.T. compound (Sakura). Embedded tissues were sectioned on a cryostat with a 14 µm step for embryonic stages and 50 µm for postnatal brains. In situ hybridization on sections and whole-mount preparations was performed as previously described [65]. *In situ* hybridization probes used in this study were mouse *Bhlhb5*, *Cdh8*, *CoupTF1*, *Emx2*, *Erm*, *Fgf8*, *Fgf15, Fgf17*, *Gli3*, *Lmo4*, *Msx1*, *Pax6*, *p73*, *Pea3*, *Reln*, *RORβ*, *Shh*, *Sp8*, *Wnt3a*, *Wnt7b*, and *Wnt8b*. For BrdU experiments, sections were incubated for 10 min in 4% PFA, 0.1M PB, rinsed 3 times in PBS and permeabilized with 4N HCl for 5 min. Immunohistochemistry on sections was performed as previously described [65]. Primary antibodies were rabbit anti-β-galactosidase (Rockland; 1∶1000), G10 mouse anti-Reelin (Calbiochem; 1∶1000), goat anti-p73 (Santa Cruz; 1∶200), rabbit anti-Tbr1 (Chemicon; 1∶1000), rabbit anti-Tbr2 (Chemicon; 1∶2000), rabbit anti-PH3 (Upsdate; 1∶500), rabbit anti-GFP (Molecular Probes; 1∶1000), rabbit anti-Dbx1 (gift of S. Morton and T.M. Jessell, 1∶10000), chicken anti-β-galactosidase (AbCam; 1∶2000), mouse anti-Mash1 (BD Pharmingen; 1∶100), mouse anti-Ngn2 (gift of D.J. Anderson; 1∶10), goat anti-Sp8 (Santa Cruz; 1∶8000), mouse anti-Pax6 (DSHB; 1∶50), and rat anti-BrdU (Accurate Chemical; 1∶400). All fluorescent secondary antibodies were purchased from Jackson ImmunoResearch. Tbr1 antibodies were also detected with biotinylated secondary antibodies using the Elite ABC kit (Vector). TUNEL staining was performed according to the manufacturer's protocol (Roche). The triple immunohistochemistry using rabbit anti-Tbr1 and rabbit anti-Dbx1 in Figure 1 was performed with Zenon Alexa Fluor 647 Rabbit IgG according to the manufacturer′s protocol (Invitrogen).

### Images Acquisition

Whole mount brain pictures were acquired using a digital camera (Zeiss Axiocam HRc) coupled to a binocular lens (Leica MZFLIII), brightfield pictures of telencephalon sections using a colour camera (Zeiss Axiocam HRc) coupled to a Zeiss Axiovert 200 microscope, and immunofluorescence pictures using an inverted confocal microscope (Leica TCS SP5 AOBS Tandem resonant Scanner). Most images in brightfield are composites and were acquired using an AxioVision 4.6 software, options Mosaix and Tiling, which automatically acquires multiple images on the same specimen and reconstructs the final image.

### Data Collection and Statistical Analysis

For all experiments, results have been obtained from at least three pairs of control and mutant littermates. Quantification of cell numbers was carried out at several levels along the rostrocaudal axis, namely L1, a rostral level where Dbx1 protein and mRNA were not detected; L2, a level where Dbx1 protein and mRNA are detectable and L3, a caudal level (at the choroid plexus level). On each section, the number of cells was counted in boxes that were placed over the DM, D, DL and L pallium in region-matched control and mutant sections. First, a box for the DM region was defined as the dorsal half of the medial wall in between the dorsal and the ventral morphological hinges. The same plial length was then used to define matched size boxes for the three other regions. All measurements realized in these boxes were normalized in number of cells per 100 µm (measured at the pial surface for Reln, p73, Tbr1 and Tbr2 and at the ventricular surface for PH3 experiments). Lengths and sizes measurements were done using Image J Software. For all quantifications, normal distribution was confirmed and unpaired, two-tailed *t* test on group means were performed for statistical analysis, using Microsoft Excel software.

### Cortex Dissection and Fluorescent Activated Cell Sorting

E12.5 *Dbx1^CRE^;ROSA26^YFP^* embryos were selected using a fluorescent stereomicroscope. DM and DL cortical regions at L1 and L2 levels were dissected as shown in Figure S6 by separating them at the dorsal hinge. The percentage of septum and PSB *Dbx1*-derived CR cells in each sample was estimated using immunohistochemistry and in situ hybridization for Reln and p73 on matched sections, as for Figure 1 and Figure 2, and Calretinin [20]. Explants were kept in cold Hank's (Invitrogen) and treated with 0.25% trypsin (Invitrogen) at 37°C for 5 min. After digestion, 0.1% FBS serum (Invitrogen) was added and cell suspension was obtained by mechanical dissociation. Dissociated cells were filtered through a 50 µm nylon mesh filter (celltrics Partec) and propidium iodide (PI) (0.1 µg/mL, final concentration) was added to cells immediately prior to analyzing and sorting. Analysis and cell sorting were performed using an Influx 500 cell sorter (Cytopeia, BD Biosciences since 2008, San Jose, CA, USA). Yellow fluorescent protein (YFP) and PI were excited with solid-state laser 488 nm, 200 mw (Coherent sapphire) and their emission signals were detected using a 528/38 nm band pass (BP) filter and a 610/20 BP, respectively. Fluorescence data were displayed on four-decade log scales. Sorts were performed at low pressure (15 PSI) with a 100 micron nozzle. Positive and negative YFP cell populations were collected simultaneously from the same sample, excluding dead cells by gating on negative red fluorescence (PI^−^) regions. YFP^−^ embryos were used as control for fluorescence. The purity of the YFP^+^ sorted cells using the established windowing level was confirmed by analysis under a fluorescent microscope and estimated to be above 98%. An average of 6 090 YFP^+^ cells for DL and 22 150 for DM were obtained from 10 embryos dissected from 3 litters.

### RNA Preparation, Array Hybridization, and Microarray Analysis

Each experimental condition was tested in duplicates. Total RNA was extracted from YFP^+^ cells using the RNeasy mini Kit (Qiagen) following the manufacturer's protocol. Biotinylated cRNAs were prepared from 3 to 5 ng of total RNA using the GeneChip Expression 3′ Amplification Two-Cycle Target Labeling and Control Reagents, according to the manufacturer's instructions (Affymetrix, Santa Clara, CA, USA). Following fragmentation, cRNAs were hybridized for 16 hours at 45°C on GeneChip Mouse Genome 430 2.0 arrays, interrogating over 39 000 transcripts. Each microarray was then washed and stained using the EukGE-WS2v5_450 protocol on a GeneChip fluidics station 450 and further scanned with a GeneChip Scanner 3000 7G. Image processing and analyses were performed using GeneChip Operating Software (GCOS) version 1.4. Absolute and comparison analyses between experimental conditions were conducted using the statistics-based Affymetrix algorithms MAS-5.0 [67],[68] with default settings and global scaling as normalization method. The trimmed mean target intensity of each chip was arbitrarily set to 100 and were selected the genes which showed an “increase” or “marginal increase” difference as the result of this statistical analysis. Additional data analysis was performed using MatLab (The MathWorks, USA) and Mev v4.4 (TM4 microarrays software suite) [69].

### Quantitative Real-Time PCR

20 ng of RNA extracted from YFP^+^ cells were used for cDNA synthesized with the SuperScript VILO cDNA Synthesis Kit (Invitrogen), following the manufacturer's instructions. Real-time PCR was carried out on a Roche LightCycler according to the manufacturer's instructions for the SYBRGreen detection kit. Primers were designed using PrimerBank [70] and Primer3 [71]. The primers were verified for specificity with Primer-Blast from NCBI. Expression of each gene was calculated relative to that of the mRNA for the ribosomal protein rpS17 [72] in the same sample for the DM and DL cell types and in three independent experiments. Relative quantifications of gene expression were calculated as described by Livak and Schmittgen [73]. The PCR efficiency for each primer pair was estimated with the LightCycler software using a calibration dilution curve for each primer set.

## Supporting Information

Figure S1 **Molecular profile of CR subtypes according to their localization along the RC and DV axis.** In situ hybridization with *Reln* (A, C–E) and *p73* (B, F–H) RNA probes were performed on E11.0 (A and B) and E11.5 coronal sections (C–H) of *Dbx1^nlsLacZ/+^* telencephalons. (A_ii_) and (B_ii_) are high magnifications of (A_i_) and (B_i_) in the lateral region of the telencephalon, showing that this region is populated exclusively by *Reln^+^* cells at early stages, corresponding to PSB-derived CR cells. *Reln* and *p73* cell numbers for each rostrocaudal level, namely L1 (C and F), L2 (D and G) and L3 (E and H) are quantified in (I), (J) and (K), respectively. Histograms represent mean ± s.e.m. In L1, *p73^+^* cells number is higher than that of *Reln^+^* cells in DM regions, whereas there are more *Reln^+^* cells in D, DL and L territories (*n* = 4). In L2 and L3, there are more *p73^+^* than *Reln^+^* cells in DM and D territories, respectively, whereas the L region contains more *Reln^+^* than *p73^+^* cells (*n* = 4). *P<0.05. Scale bars: 200 µm (A_i_, B_i_ and C–H) and 100 µm (A_ii_ and B_ii_).
(4.45 MB TIF)

Figure S2 **Specific ablation of septum** ***Dbx1*** **-derived CR cells using** ***Emx1^iresCre^*** **mice.** (A and B) At L2 levels, sections of E11.5 *ROSA26^YFP^;Emx1^iresCre^* embryos were immunostained for Dbx1 and YFP. In the septum (A_i_, high magnification in A_ii_) dorsal Dbx1^+^ cells are YFP^+^ (white arrowheads) whereas ventral Dbx1^+^ cells are YFP^−^ (black arrowheads), although both are of pallial origin (Tbr1^+^, see Figure 1F). High magnification of the PSB (B_ii_, white box in B_i_) shows that there is no colabeling of YFP with Dbx1 (black arrowheads). (C and D) TUNEL (green) staining on E11.5 sections of control (C) and *Dbx1^DTA^;Emx1^iresCre^* embryos (D). There are no TUNEL^+^ cells in the DM region in control (C_ii_) and mutant embryos (D_ii_). TUNEL^+^ cells are detected at the septum of mutant embryos (D_iii_) but not in controls (C_iii_). Some TUNEL^+^ cells are also detected in the mantle zone of the PSB in mutant embryos (D_iv_) but do not correspond to CR cells since no loss of *Reln* was observed in the lateral pallium. (E–H) Immunofluorescence using Dbx1 antisera showing that at the septum dorsal Dbx1^+^ cells are deleted in mutant embryos (F) compared to control animals (E) whereas ventral Dbx1^+^ cells are not (compare G and H). In situ hybridization for *Reln* (I, K, quantified in M) and *p73* (J, L, quantified in N) at caudal levels shows that there is no significant difference (*n* = 6) in the numbers of *Reln^+^* and *p73^+^* cells between E11.5 control (I and J) and *Dbx1^DTA^;Emx1^iresCre^* embryos (K and L). Quantifications of the numbers of *Reln^+^* and *p73^+^* cells in L1 (O) and L2 (P) DM regions of E12.5 control (white bars) and *Dbx1^DTA^;Emx1^iresCre^* embryos (black bars) (*n* = 3). *P<0.05. Scale bars: 200 µm (C_i_ and D_i_), 100 µm (C_ii_–C_iv_, D_ii_–D_iv_ and I–L), 50 µm (A_i_, B_i_, G, and H) and 20 µm (A_ii_, B_ii_, E,and F).
(2.50 MB TIF)

Figure S3 **Changes in regionalization markers gradients at E11.5 and E12.5.** In situ hybridization on coronal sections of E11.5 control (A, C, and E) and *Dbx1^DTA^;Emx1^iresCre^* (B, D, and F) telencephalons using *Emx2* RNA probe (*n* = 4). (E) and (F) are pseudocolors high magnifications of DM/D regions in (A) and (B), respectively. In situ hybridization for *Emx2* (G and H) and *CoupTF1* (I and J) were performed on whole mount brains of E12.5 control (G and I) and *Dbx1^DTA^;Emx1^iresCre^* (H and J) embryos (*n* = 3). *Emx2* expression is extended rostrally at medial levels of the mutant telencephalons (compare [G] and [H]). Black arrowheads represent the limits of domains of high expression in controls and red arrowheads indicate the shift observed in mutant embryos. Rostromedial low *CoupTF1* expression domain is expanded in mutant embryos (J), compared to controls (I). Coronal sections of E12.5 control (K–M) and *Dbx1^DTA^;Emx1^iresCre^* (N–P) embryos were immunostained with Pax6 (K, M, N, and P) and Sp8 (L, M, O, and P). Left to this panel is represented a dorsal view of E12.5 brains. The red dashed line indicates the RC level of sections shown in (K–P). R, rostral; C, caudal. Scale bars: 1mm (G–J), 200 µm (A–B) and 100 µm (C–F and K–P).
(2.43 MB TIF)

Figure S4 **The expression of signaling molecules and dorsoventral patterning are not affected after septum-derived CR cells ablation.** (A–F) In situ hybridization with *Shh* (A–B), *Msx1* (C–D) and *Gli3* (E–F) RNA probes on E11.5 control (A, C, and E) and *Dbx1^DTA^;Emx1^iresCre^* (B, D, and F) coronal sections showing that the expression domains are not altered in *Dbx1^DTA^;Emx1^iresCre^* embryos. Immunohistochemistry on sections of E11.5 control (G, I, K, and M) and *Dbx1^DTA^;Emx1^iresCre^* embryos (H, J, L, and N) at L2 (G,H,K,L) and at more caudal (taenia tecta, I,J,M,N) levels of the septum. Most of Dbx1^+^ cells are absent in *Dbx1^DTA^;Emx1^iresCre^* embryos (H and L) compared to controls (G and K) at the rostral septum (L2 level). Boundaries of Ngn2 (I and J, white arrowheads) and Mash1 (M and N, white arrowheads) are similar in control and mutant embryos (*n* = 3). Scale bars: 200 µm (A–F) and 100 µm (G–N).
(5.84 MB TIF)

Figure S5 **Ablation of septum-derived CR cells leads to changes in the relative size of cortical areas without affecting cortical lamination.** (A and B) P0 brains stained with *Cdh8* RNA probe, in control (A) and *Dbx1^DTA^;Emx1^iresCre^* (B) animals. Dorsal views show that the rostral *Cdh8* expression domain is expanded towards the caudal and lateral regions in mutant animals (B). Quantifications of overall cortical area size (C) and relative sizes of motor and visual areas based on the *Cdh8* staining (D). Histograms represent mean ± s.e.m. (*n* = 5, *P<0.05). (E–H) P0 brains stained with *Cdh8* (E and F) and *Lmo4* (G and H) RNA probes, in control (E and G) and *Dbx1^DTA^;deltaNp73* (F and H) animals showing an increase in motor area size as in *Dbx1^DTA^;Emx1^iresCre^* animals (*n* = 3). Nissl staining was performed on P8 control (I and M), *Dbx1^DTA^;Emx1^iresCre^* (J) and *Dbx1^DTA^;deltaNp73* (N) brains showing that lamination is unaltered in mutant animals (see also Tbr1 staining in [K] and [L] and *RORβ*, *Cdh8* and *Lmo4* in Figure 6). (O–R) In situ hybridization with *Reln* (O and P) and *p73* (Q and R) RNA probes at L1 levels of E11.5 control (O and Q) and *Dbx1^DTA^;deltaNp73* (P and R) embryos, showing a decrease in *Reln* and *p73* in rostrodorsal regions. Compensation in the ventromedial region by young hem-derived CR cells (Reln^−^/p73^+^) has already occurred in these embryos. (O_ii_,O_iii_, P_ii_,P_iii_, Q_ii_,Q_iii_ and R_ii_,R_iii_) are high magnifications of boxed domains in (O_i_, P_i_, Q_i_, and R_i_), in dorsolateral and dorsomedial regions, respectively. (S and T) In situ hybridization with *Cdh8* RNA probe on sagittal sections of P8 brains at medial levels. The rostral *Cdh8* expression domain is reduced (red arrowhead) whereas *Cdh8* expression domains in the visual (blue arrowhead, T) and retrosplenial areas (brackets) are shifted towards the rostral region in mutant brains (T) compared to controls (S) (*n* = 3). Scale bars: 1 mm (A–B, E–N, and S–T), 200 µm (O_i_, P_i_, Q_i_ and R_i_) and 50 µm (O_ii_, O_iii_, P_ii_, P_iii_, Q_ii_, Q_iii_, R_ii_, and R_iii_).
(3.52 MB TIF)

Figure S6 **Dissection and flow cytometry analysis of** ***Dbx1^CRE^;ROSA26^YFP^*** **cells.** (A–C) E12.5 coronal sections of *Dbx1^CRE^;ROSA26^YFP^* embryos were immunostained with YFP and Reln antibodies. The white dashed lines delimit the pallial regions which were dissected for purification. (B) and (C) are high magnifications of white boxes in (A) DM and DL regions. (A) is a composite of two images acquired on the dorsal and ventral telencephalon. (D–F) Representative histograms depict log fluorescence intensity on the « X axis » and events on the «Y axis » for control (D), DM (E) and DL (F) samples. Major peaks represent background log fluorescence relative to control samples whereas R regions represent labeled YFP cells. (G–I) Bi-parametric graphs show the gated R region used for sorting YFP^+^ cells. Dead cells were excluded simultaneously from sorted cells using gated negative PI regions. No positive cells appear in R in the control sample (G) and positive YFP regions are well separated from negative ones in DM (H) and DL (I) samples. The number of cells is shown by the number of dots where each dot represents a single cell. Analysis was normalized on 13 000 cells after exclusion of cell aggregates and dead cells. Scale bars: 100 µm (A) and 10 µm (B and C).
(1.55 MB TIF)

Table S1 **List of gene expression profiles of DM and DL FACS-sorted** ***Dbx1*** **-derived cells.** Affymetrix probeset ID, gene name, signal value in DM and DL, ratios DM/DL and DL/DM, *p* values are listed for each gene. * represent values called “Absent” in the Affymetrix analysis. NC: no change.
(0.13 MB DOC)

Table S2 **Relative expression levels of Fgfs and Wnts in DM and DL FACS-sorted** ***Dbx1*** **-derived cells.** In the table are listed the values of the relative expression of the Fgfs and Wnts genes in YFP^+^ cells of DM with respect to DL pallial regions, as well as the standard deviations and the *p* values of the *t*-test. In the last two columns are listed the expression values of the different genes, normalized to the reference gene rpS17, in DM and DL, respectively.
(0.03 MB DOC)

## Abbreviations

CR: Cajal-Retzius
D: dorsal
DL: dorsolateral
DM: dorsomedial
L: lateral
MZ: marginal zone
PSB: pallial-subpallial boundary
RC: rostrocaudal
TFs: transcription factors
VZ: ventricular zone
