# Supplementary figures and images for: A Novel Role for Dbx1-Derived Cajal-Retzius Cells in Early Regionalization of the Cerebral Cortical Neuroepithelium
Source: PLoS Biol. 2010 Jul 27;8(7):e1000440. doi: 10.1371/journal.pbio.1000440 (PMC2910656; doi:10.1371/journal.pbio.1000440)

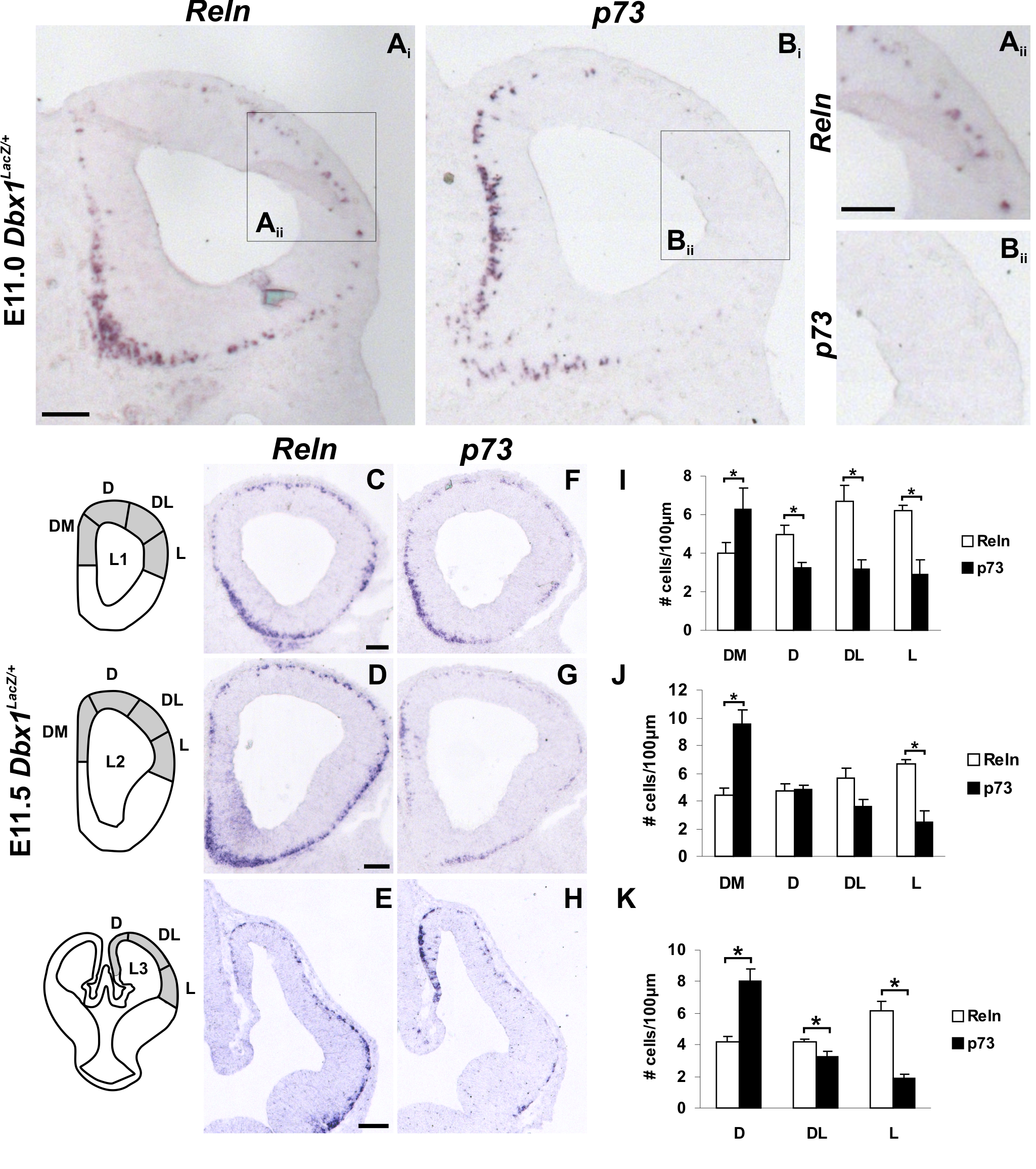

Supplement: Figure S1 — Molecular profile of CR subtypes according to their localization along the RC and DV axis. In situ hybridization with Reln (A, C–E) and p73 (B, F–H) RNA probes were performed on E11.0 (A and B) and E11.5 coronal sections (C–H) of Dbx1nlsLacZ/+ telencephalons. (Aii) and (Bii) are high magnifications of (Ai) and (Bi) in the lateral region of the telencephalon, showing that this region is populated exclusively by Reln+ cells at early stages, corresponding to PSB-derived CR cells. Reln and p73 cell numbers for each rostrocaudal level, namely L1 (C and F), L2 (D and G) and L3 (E and H) are quantified in (I), (J) and (K), respectively. Histograms represent mean ± s.e.m. In L1, p73+ cells number is higher than that of Reln+ cells in DM regions, whereas there are more Reln+ cells in D, DL and L territories (n = 4). In L2 and L3, there are more p73+ than Reln+ cells in DM and D territories, respectively, whereas the L region contains more Reln+ than p73+ cells (n = 4). *P<0.05. Scale bars: 200 µm (Ai, Bi and C–H) and 100 µm (Aii and Bii). (4.45 MB TIF) [file pbio.1000440.s001.tif]

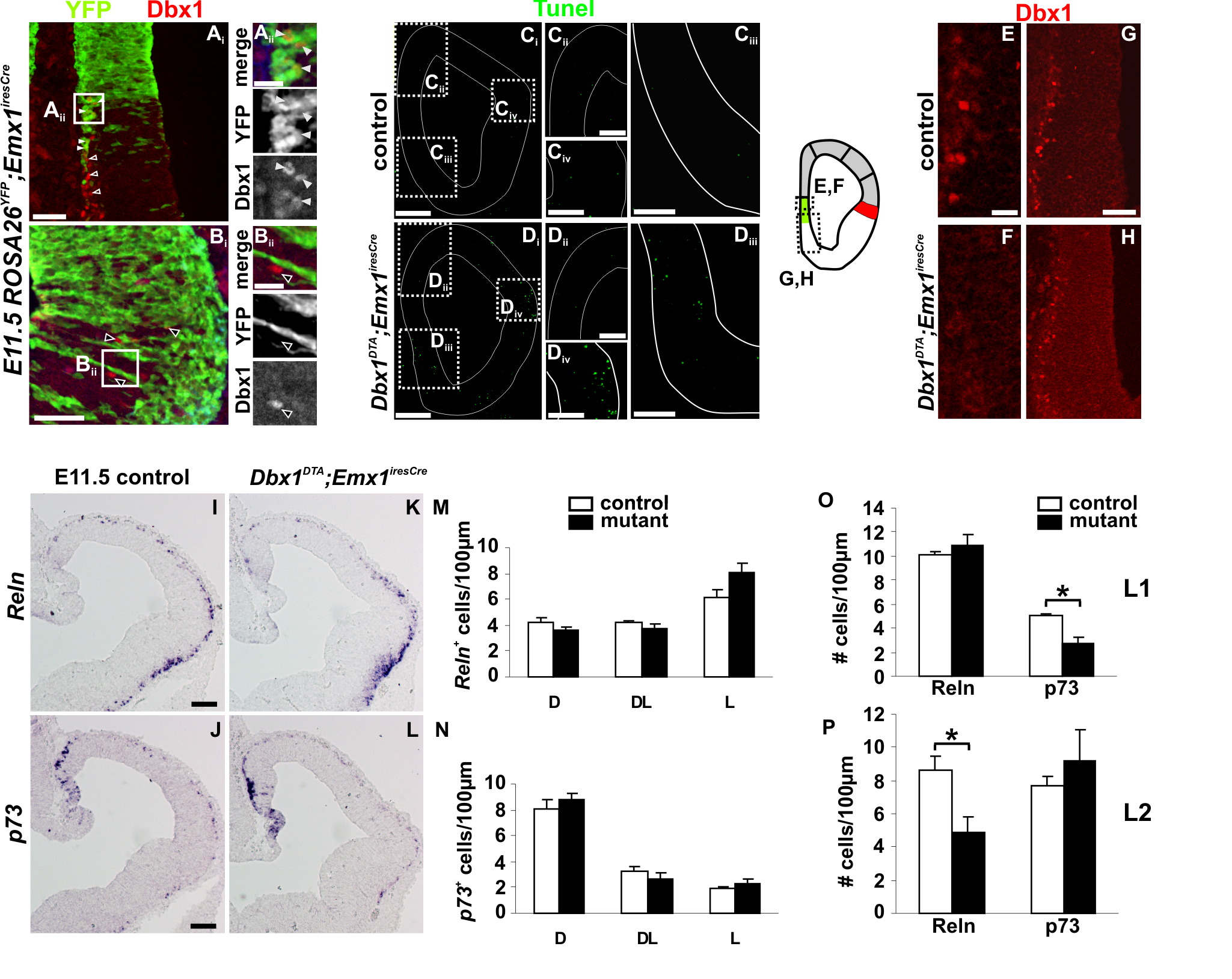

Supplement: Figure S2 — Specific ablation of septum Dbx1 -derived CR cells using Emx1iresCre mice. (A and B) At L2 levels, sections of E11.5 ROSA26YFP;Emx1iresCre embryos were immunostained for Dbx1 and YFP. In the septum (Ai, high magnification in Aii) dorsal Dbx1+ cells are YFP+ (white arrowheads) whereas ventral Dbx1+ cells are YFP− (black arrowheads), although both are of pallial origin (Tbr1+, see Figure 1F). High magnification of the PSB (Bii, white box in Bi) shows that there is no colabeling of YFP with Dbx1 (black arrowheads). (C and D) TUNEL (green) staining on E11.5 sections of control (C) and Dbx1DTA;Emx1iresCre embryos (D). There are no TUNEL+ cells in the DM region in control (Cii) and mutant embryos (Dii). TUNEL+ cells are detected at the septum of mutant embryos (Diii) but not in controls (Ciii). Some TUNEL+ cells are also detected in the mantle zone of the PSB in mutant embryos (Div) but do not correspond to CR cells since no loss of Reln was observed in the lateral pallium. (E–H) Immunofluorescence using Dbx1 antisera showing that at the septum dorsal Dbx1+ cells are deleted in mutant embryos (F) compared to control animals (E) whereas ventral Dbx1+ cells are not (compare G and H). In situ hybridization for Reln (I, K, quantified in M) and p73 (J, L, quantified in N) at caudal levels shows that there is no significant difference (n = 6) in the numbers of Reln+ and p73+ cells between E11.5 control (I and J) and Dbx1DTA;Emx1iresCre embryos (K and L). Quantifications of the numbers of Reln+ and p73+ cells in L1 (O) and L2 (P) DM regions of E12.5 control (white bars) and Dbx1DTA;Emx1iresCre embryos (black bars) (n = 3). *P<0.05. Scale bars: 200 µm (Ci and Di), 100 µm (Cii–Civ, Dii–Div and I–L), 50 µm (Ai, Bi, G, and H) and 20 µm (Aii, Bii, E,and F). (2.50 MB TIF) [file pbio.1000440.s002.tif]

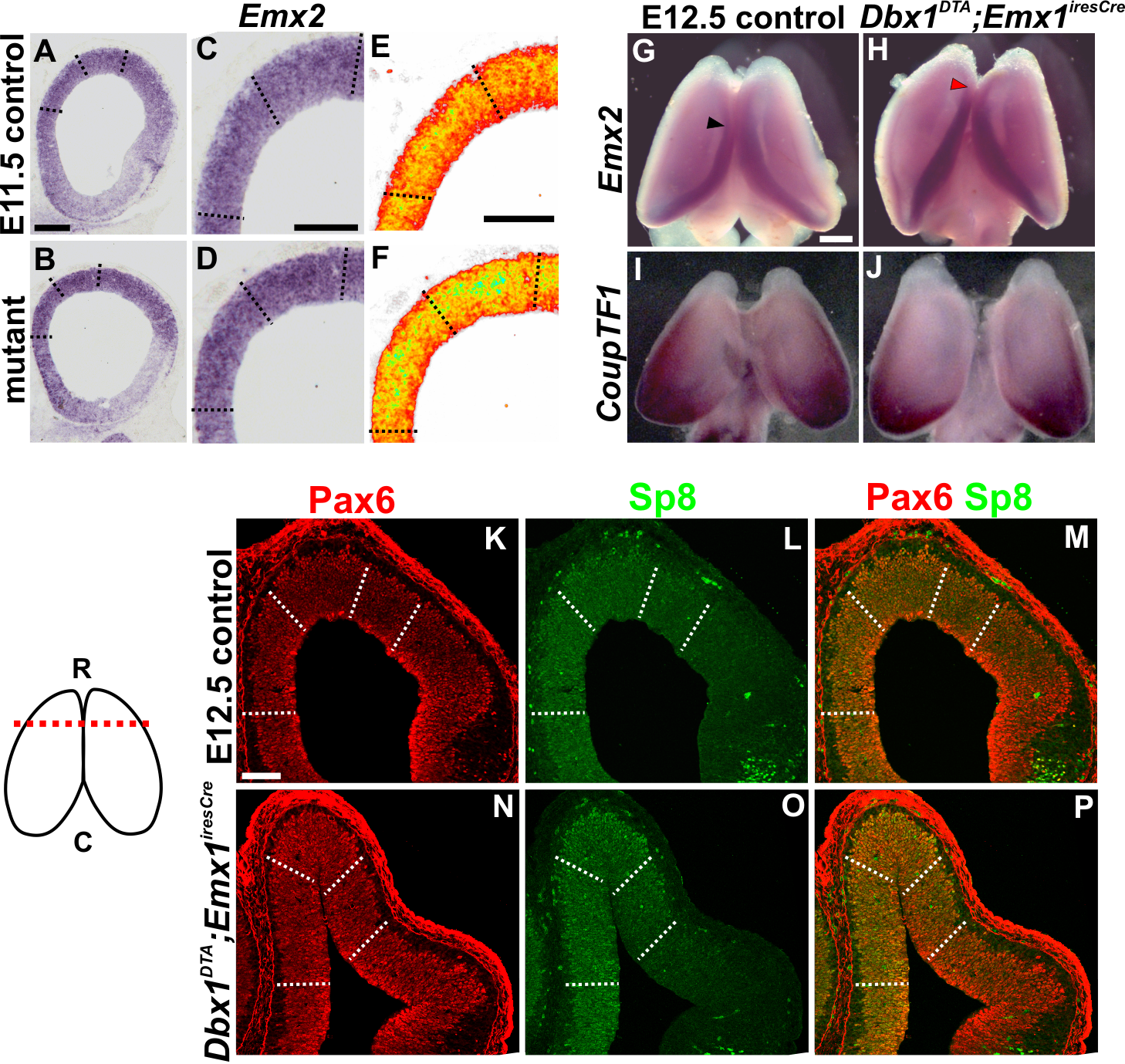

Supplement: Figure S3 — Changes in regionalization markers gradients at E11.5 and E12.5. In situ hybridization on coronal sections of E11.5 control (A, C, and E) and Dbx1DTA;Emx1iresCre (B, D, and F) telencephalons using Emx2 RNA probe (n = 4). (E) and (F) are pseudocolors high magnifications of DM/D regions in (A) and (B), respectively. In situ hybridization for Emx2 (G and H) and CoupTF1 (I and J) were performed on whole mount brains of E12.5 control (G and I) and Dbx1DTA;Emx1iresCre (H and J) embryos (n = 3). Emx2 expression is extended rostrally at medial levels of the mutant telencephalons (compare [G] and [H]). Black arrowheads represent the limits of domains of high expression in controls and red arrowheads indicate the shift observed in mutant embryos. Rostromedial low CoupTF1 expression domain is expanded in mutant embryos (J), compared to controls (I). Coronal sections of E12.5 control (K–M) and Dbx1DTA;Emx1iresCre (N–P) embryos were immunostained with Pax6 (K, M, N, and P) and Sp8 (L, M, O, and P). Left to this panel is represented a dorsal view of E12.5 brains. The red dashed line indicates the RC level of sections shown in (K–P). R, rostral; C, caudal. Scale bars: 1mm (G–J), 200 µm (A–B) and 100 µm (C–F and K–P). (2.43 MB TIF) [file pbio.1000440.s003.tif]

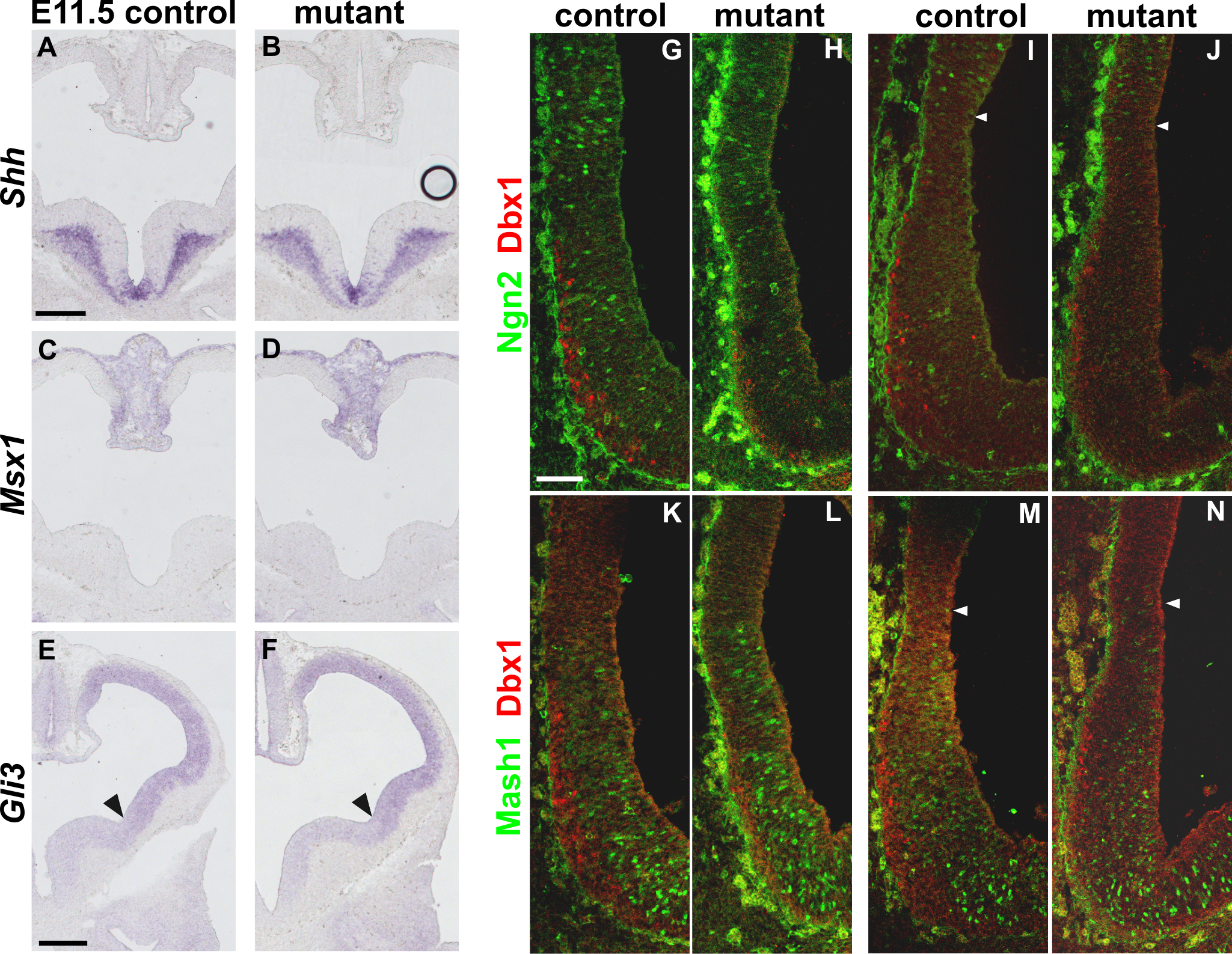

Supplement: Figure S4 — The expression of signaling molecules and dorsoventral patterning are not affected after septum-derived CR cells ablation. (A–F) In situ hybridization with Shh (A–B), Msx1 (C–D) and Gli3 (E–F) RNA probes on E11.5 control (A, C, and E) and Dbx1DTA;Emx1iresCre (B, D, and F) coronal sections showing that the expression domains are not altered in Dbx1DTA;Emx1iresCre embryos. Immunohistochemistry on sections of E11.5 control (G, I, K, and M) and Dbx1DTA;Emx1iresCre embryos (H, J, L, and N) at L2 (G,H,K,L) and at more caudal (taenia tecta, I,J,M,N) levels of the septum. Most of Dbx1+ cells are absent in Dbx1DTA;Emx1iresCre embryos (H and L) compared to controls (G and K) at the rostral septum (L2 level). Boundaries of Ngn2 (I and J, white arrowheads) and Mash1 (M and N, white arrowheads) are similar in control and mutant embryos (n = 3). Scale bars: 200 µm (A–F) and 100 µm (G–N). (5.84 MB TIF) [file pbio.1000440.s004.tif]

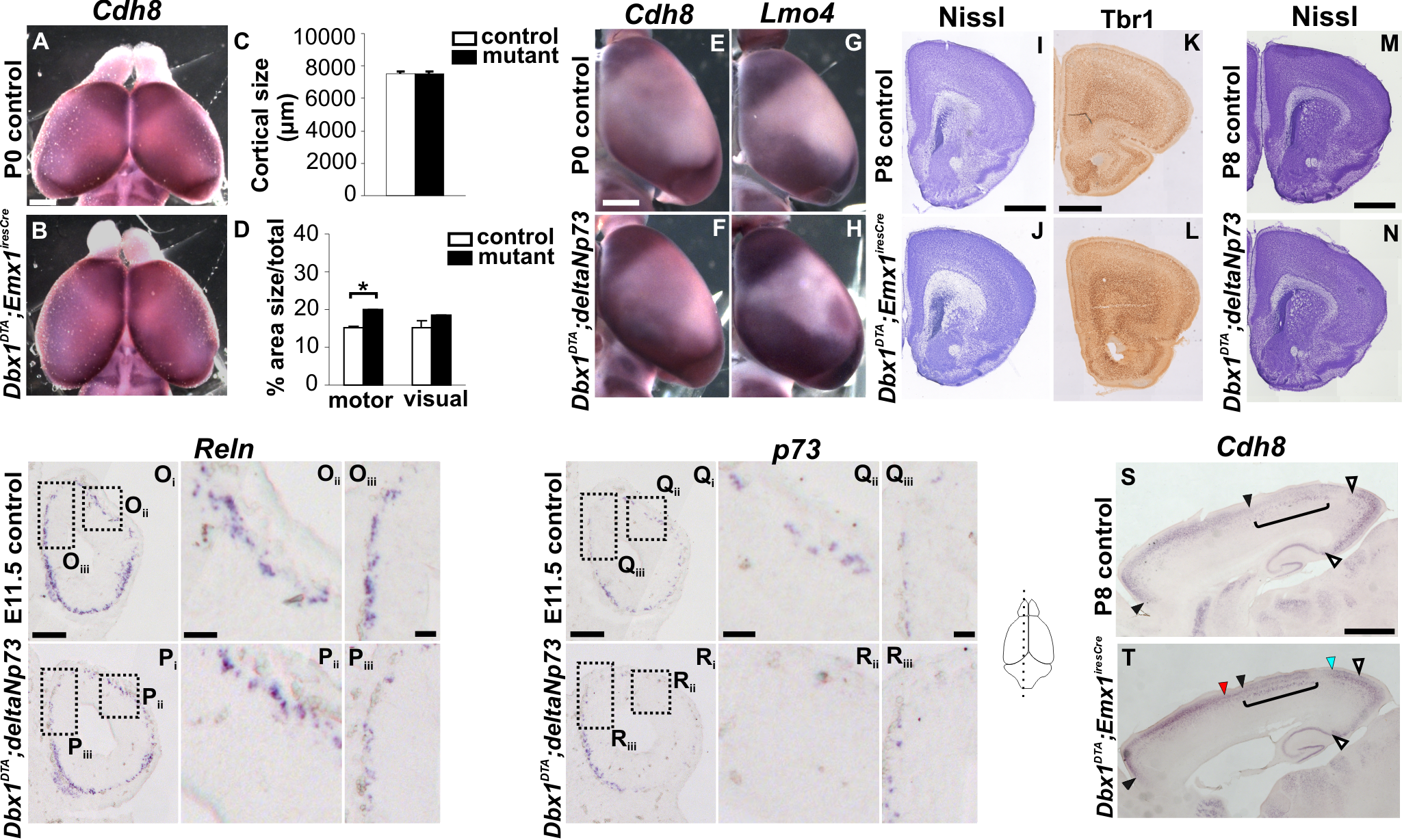

Supplement: Figure S5 — Ablation of septum-derived CR cells leads to changes in the relative size of cortical areas without affecting cortical lamination. (A and B) P0 brains stained with Cdh8 RNA probe, in control (A) and Dbx1DTA;Emx1iresCre (B) animals. Dorsal views show that the rostral Cdh8 expression domain is expanded towards the caudal and lateral regions in mutant animals (B). Quantifications of overall cortical area size (C) and relative sizes of motor and visual areas based on the Cdh8 staining (D). Histograms represent mean ± s.e.m. (n = 5, *P<0.05). (E–H) P0 brains stained with Cdh8 (E and F) and Lmo4 (G and H) RNA probes, in control (E and G) and Dbx1DTA;deltaNp73 (F and H) animals showing an increase in motor area size as in Dbx1DTA;Emx1iresCre animals (n = 3). Nissl staining was performed on P8 control (I and M), Dbx1DTA;Emx1iresCre (J) and Dbx1DTA;deltaNp73 (N) brains showing that lamination is unaltered in mutant animals (see also Tbr1 staining in [K] and [L] and RORβ, Cdh8 and Lmo4 in Figure 6). (O–R) In situ hybridization with Reln (O and P) and p73 (Q and R) RNA probes at L1 levels of E11.5 control (O and Q) and Dbx1DTA;deltaNp73 (P and R) embryos, showing a decrease in Reln and p73 in rostrodorsal regions. Compensation in the ventromedial region by young hem-derived CR cells (Reln−/p73+) has already occurred in these embryos. (Oii,Oiii, Pii,Piii, Qii,Qiii and Rii,Riii) are high magnifications of boxed domains in (Oi, Pi, Qi, and Ri), in dorsolateral and dorsomedial regions, respectively. (S and T) In situ hybridization with Cdh8 RNA probe on sagittal sections of P8 brains at medial levels. The rostral Cdh8 expression domain is reduced (red arrowhead) whereas Cdh8 expression domains in the visual (blue arrowhead, T) and retrosplenial areas (brackets) are shifted towards the rostral region in mutant brains (T) compared to controls (S) (n = 3). Scale bars: 1 mm (A–B, E–N, and S–T), 200 µm (Oi, Pi, Qi and Ri) and 50 µm (Oii, Oiii, Pii, Piii, Qii, Qiii, Rii, and Riii). (3.5 [file pbio.1000440.s005.tif]

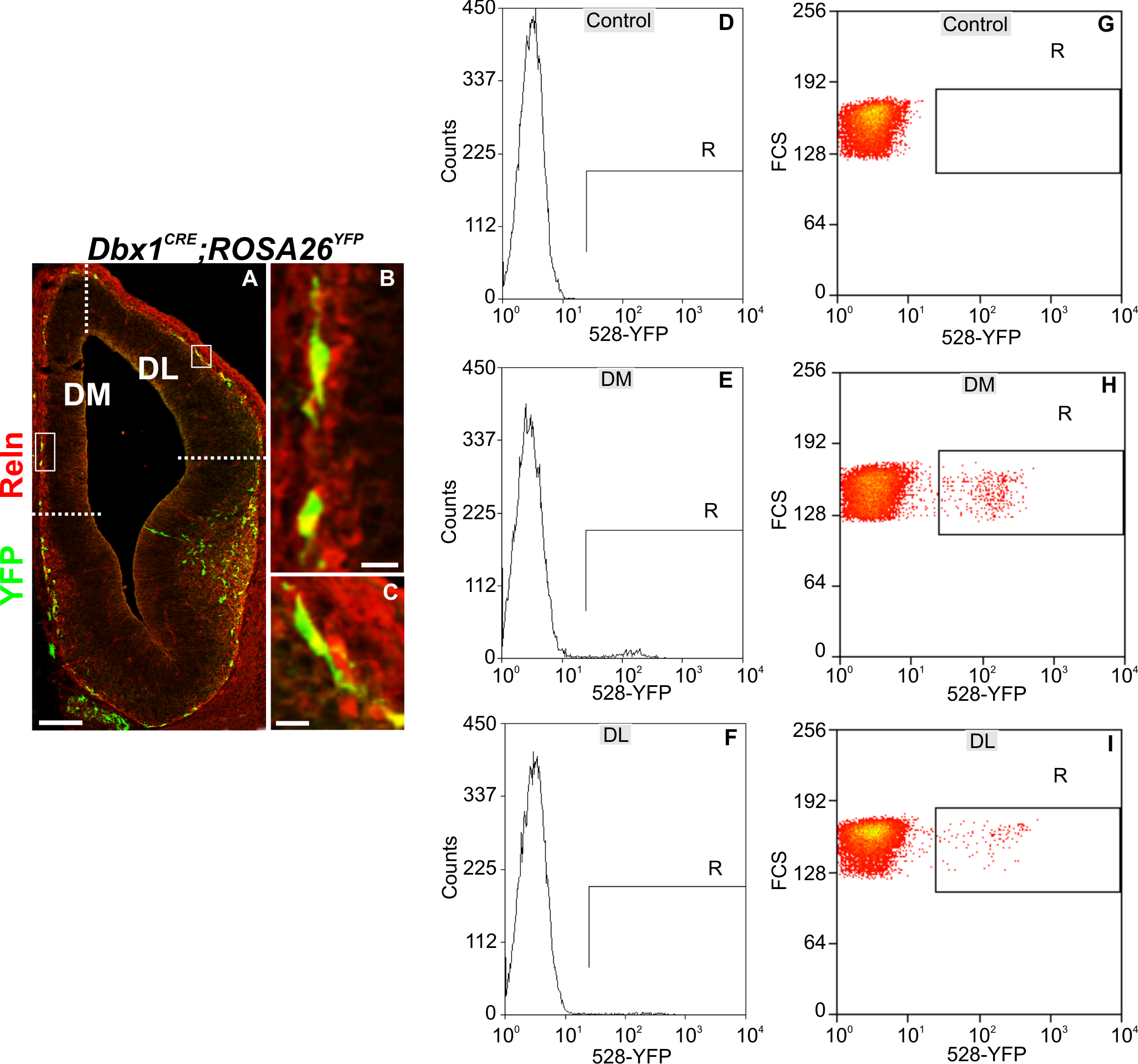

Supplement: Figure S6 — Dissection and flow cytometry analysis of Dbx1CRE;ROSA26YFP cells. (A–C) E12.5 coronal sections of Dbx1CRE;ROSA26YFP embryos were immunostained with YFP and Reln antibodies. The white dashed lines delimit the pallial regions which were dissected for purification. (B) and (C) are high magnifications of white boxes in (A) DM and DL regions. (A) is a composite of two images acquired on the dorsal and ventral telencephalon. (D–F) Representative histograms depict log fluorescence intensity on the « X axis » and events on the «Y axis » for control (D), DM (E) and DL (F) samples. Major peaks represent background log fluorescence relative to control samples whereas R regions represent labeled YFP cells. (G–I) Bi-parametric graphs show the gated R region used for sorting YFP+ cells. Dead cells were excluded simultaneously from sorted cells using gated negative PI regions. No positive cells appear in R in the control sample (G) and positive YFP regions are well separated from negative ones in DM (H) and DL (I) samples. The number of cells is shown by the number of dots where each dot represents a single cell. Analysis was normalized on 13 000 cells after exclusion of cell aggregates and dead cells. Scale bars: 100 µm (A) and 10 µm (B and C). (1.55 MB TIF) [file pbio.1000440.s006.tif]
