# Supplementary material for: A Novel Role for Dbx1-Derived Cajal-Retzius Cells in Early Regionalization of the Cerebral Cortical Neuroepithelium
Source: PLoS Biol. 2010 Jul 27;8(7):e1000440. doi: 10.1371/journal.pbio.1000440 (PMC2910656; doi:10.1371/journal.pbio.1000440)
Supplement: Table S1 — List of gene expression profiles of DM and DL FACS-sorted Dbx1 -derived cells. Affymetrix probeset ID, gene name, signal value in DM and DL, ratios DM/DL and DL/DM, p values are listed for each gene. * represent values called “Absent” in the Affymetrix analysis. NC: no change. (0.13 MB DOC) [file pbio.1000440.s007.doc]

| **Probeset ID** | **Gene name** | **signal DM** | **signal DL** | **ratio DM vs DL** | **ratio DL vs DM** | ***p* value** |
| --- | --- | --- | --- | --- | --- | --- |
| **FGF signaling** |  |  |  |  |  |  |
| 1418497_at | Fgf13 | 456,5 | 755,5 | 0,604 | 1,655 | 0,00002 |
| 1418376_at | Fgf15 | 511,5 | 50,2 | 10,189 | 0,098 | 0,00002 |
| 1456239_at | Fgf17 | 650,7 | 72,3 | 9,000 | 0,111 | 0,00002 |
| 1449545_at | Fgf18 | 177,2 | 18,5 | 9,578 | 0,104 | 0,00002 |
| 1451882_a_at | Fgf8 | 53,9 | 11,1 | 4,856 | 0,206 | 0,0000242 |
| 1438718_at | Fgf9 | 67,9 | 87,3 | 0,778 | 1,286 | 0,000341 |
| 1440270_at | Fgf12 | 13,9 | 34,5 | 0,403 | 2,482 | 0,000989 |
| 1435747_at | Fgf14 | 17,4 | 31,3 | 0,556 | 1,799 | 0,000041 |
| 1438405_at | Fgf7 | 10,4***** | 12,9***** | NC | NC | 0.5 |
| **WNT signaling** |  |  |  |  |  |  |
| 1436791_at | Wnt5a | 108,4 | 31,3 | 3,463 | 0,289 | 0,0000455 |
| 1439373_x_at | Wnt5b | 132,3 | 36 | 3,675 | 0,272 | 0,0001595 |
| 1441316_at | Wnt8b | 152,4 | 40,9 | 3,726 | 0,268 | 0,00002 |
| 1420512_at | Dkk2 | 55,7 | 7,8 | 7,141 | 0,140 | 0,001364 |
| 1448201_at | Sfrp2 | 43,7 | 30,7 | 1,423 | 0,703 | 0.000046 |
| 1422093_at | Wnt3a | 7,5***** | 9,1***** | NC | NC | >0,5 |
| 1423367_at | Wnt7a | 19,3 | 215,8 | 0,089 | 11,181 | 0,000183 |
| 1420892_at | Wnt7b | 406,8 | 728,4 | 0,558 | 1,791 | 0,00002 |
| 1428136_at | Sfrp1 | 293,8 | 538,3 | 0,546 | 1,832 | 0,0000274 |
| **BMP/TGFβ signaling** |  |  |  |  |  |  |
| 1448259_at | Fstl1 | 1482,4 | 325,6 | 4,553 | 0,220 | 0,00002 |
| 1435479_at | Bmp7 | 206,1 | 92,2 | 2,235 | 0,447 | 0,0000275 |
| 1455851_at | Bmp5 | 146,4 | 92,9 | 1,576 | 0,635 | 0,0000649 |
| 1450923_at | Tgfb2 | 146,8 | 35,4 | 4,147 | 0,241 | 0,0000135 |
| 1421365_at | Fst | 56,5 | 110,35 | 0,512 | 1,953 | 0,0000257 |
| 1450421_at | Tgfa | 14,7***** | 13,1***** | NC | NC | 0.5 |

| **Probeset ID** | **Gene name** | **signal DM** | **signal DL** | **ratio DM vs DL** | **ratio DL vs DM** | ***p* value** |
| --- | --- | --- | --- | --- | --- | --- |
| **Other secreted molecules** |  |  |  |  |  |  |
| 1416006_at | Mdk | 1495,4 | 444,7 | 3,363 | 0,297 | 0,00002 |
| 1448254_at | Ptn | 1230,6 | 333,3 | 3,692 | 0,271 | 0,0000235 |
| 1415855_at | Kitl | 847,1 | 488,4 | 1,734 | 0,577 | 0,0000935 |
| 1427100_at | Metrn | 344,1 | 77,7 | 4,429 | 0,226 | 0,00002 |
| 1458140_at | Slit2 | 401,1 | 93,9 | 4,272 | 0,234 | 0,00002 |
| 1436869_at | Shh | 176,1 | 77,2 | NC | NC | 0,5 |
| 1454701_at | 4930503L19Rik | 176,1 | 109,6 | 1,607 | 0,622 | 0,000291 |
| 1448152_at | Igf2 | 152,5 | 45,8 | 3,330 | 0,300 | 0,0000475 |
| 1421017_at | Nrg3 | 15,1 | 34,8 | 0,434 | 2,305 | 0,000029 |
| **PP/CR markers** |  |  |  |  |  |  |
| 1449465_at | Reln | 3436,3 | 4736,4 | 0,726 | 1,378 | 0,0000335 |
| 1439627_at | Zic1 | 3894,3 | 2627,3 | 1,482 | 0,675 | 0,000036 |
| 1423424_at | Zic3 | 2585,3 | 609,5 | 4,242 | 0,236 | 0,00002 |
| 1456219_at | LOC100045988/Zic5 | 1338,6 | 294,6 | 4,544 | 0,220 | 0,00002 |
| 1456417_at | Zic4 | 834,8 | 170 | 4,911 | 0,204 | 0.00001 |
| 1435197_at | Pou3f3 | 755,6 | 2093,7 | 0,361 | 2,771 | 0,00002 |
| 1418317_at | Lhx2 | 695,5 | 1582,6 | 0,439 | 2,275 | 0,00002 |
| 1416711_at | Tbr1 | 560,3 | 1121,6 | 0,500 | 2,002 | 0,00003 |
| 1452325_at | Trp73 | 491,5 | 851,3 | 0,577 | 1,732 | 0,00002 |
| 1456258_at | Emx2 | 384,5 | 683 | 0,563 | 1,776 | 0,00002 |
| 1418494_at | Ebf2 | 407,8 | 830 | 0,491 | 2,035 | 0,00002 |
| 1450684_at | Etv1 | 186,7 | 409 | 0,456 | 2,191 | 0,00002 |
| 1441313_x_at | Lhx9 | 127 | 220,4 | 0,576 | 1,735 | 0,0000265 |
