# Supplementary material for: A Novel Role for Dbx1-Derived Cajal-Retzius Cells in Early Regionalization of the Cerebral Cortical Neuroepithelium
Source: PLoS Biol. 2010 Jul 27;8(7):e1000440. doi: 10.1371/journal.pbio.1000440 (PMC2910656; doi:10.1371/journal.pbio.1000440)
Supplement: Table S2 — Relative expression levels of Fgfs and Wnts in DM and DL FACS-sorted Dbx1 -derived cells. In the table are listed the values of the relative expression of the Fgfs and Wnts genes in YFP+ cells of DM with respect to DL pallial regions, as well as the standard deviations and the p values of the t-test. In the last two columns are listed the expression values of the different genes, normalized to the reference gene rpS17, in DM and DL, respectively. (0.03 MB DOC) [file pbio.1000440.s008.doc]

| **Gene Name** | **Ratios DM/DL** | **Standard deviation** | ***p* value** | **DM values** | **DL values** |
| --- | --- | --- | --- | --- | --- |
| Fgf8 | 0,965936329 | 0.007 | 0,02 | 0,029818404 | 0,030421393 |
| Fgf15 | 1,879045498 | 0.004 | 0,012 | 1,527534685 | 0,861488602 |
| Fgf17 | 1,986184991 | 0.006 | 0,008 | 0,287174589 | 0,144586023 |
| Fgf18 | 0,483664529 | 0.01 | 0,052 | 0,105489157 | 0,218103977 |
| Wnt3a | 0,880673949 | 0.007 | 0,015 | 0,001945969 | 0,002209636 |
| Wnt7b | 0,400718355 | 0.002 | 0,01 | 0,06938357 | 0,173147971 |
